# Transcriptomics and Metabolomics Reveal the Antagonistic Mechanism of *Bacillus velezensis* 20507 Fermentation Broth Against Fusarium Head Blight Pathogen

**DOI:** 10.3390/microorganisms14051039

**Published:** 2026-05-03

**Authors:** Siqi Yang, Ying Yang, Shihan Feng, Jianfeng Liu, Yunqing Cheng

**Affiliations:** Jilin Provincial Key Laboratory of Plant Resource Science and Green Production, Jilin Normal University, Siping 136000, China; 15943050987@163.com (S.Y.); 19843440422@163.com (Y.Y.); 15604340110@163.com (S.F.); jianfengliu1976@163.com (J.L.)

**Keywords:** *Bacillus velezensis*, Fusarium head blight (FHB), transcriptomics, metabolomics, biocontrol mechanism

## Abstract

Fusarium head blight (FHB), caused by *Fusarium graminearum*, is a devastating wheat disease leading to significant yield loss and mycotoxin contamination. This study elucidated the biocontrol mechanism of *Bacillus velezensis* 20507 fermentation broth against FHB during wheat infection. The broth exhibited strong, time-dependent antifungal activity in vitro, with optimal growth suppression (inhibition rates up to 75%) achieved using broth fermented for 3–7 days. *In planta* experiments confirmed its efficacy in alleviating disease symptoms. Employing a dual RNA-seq strategy, we analyzed the tripartite interaction between the biocontrol agent, pathogen, and wheat host. Transcriptomic analysis revealed that the broth directly suppressed the pathogen, causing 1510 differentially expressed genes (DEGs, predominantly down-regulated) and disrupting pathways related to carbohydrate metabolism and cell wall integrity. In wheat, the fermentation broth of *B. velezensis* 20507 counteracted *F. graminearum* infection by reprogramming the host transcriptome. KEGG analysis during co-inoculation showed that the broth up-regulated defense-related pathways involved in energy, hormone signaling, and cellular maintenance, while down-regulating primary metabolic pathways, indicating a resource reallocation strategy. Furthermore, transcriptomic analysis revealed that the broth alone primed the wheat defense system, and this primed state significantly enhanced the defense response upon pathogen challenge. Untargeted metabolomics identified key antimicrobial compounds, including lipopeptides and the macrolide Macrolactin A. Bioassay-guided fractionation isolated two active fractions (Fr A and Fr B) with potent antifungal activity. This integrated multi-omics study demonstrates that *B. velezensis* 20507 combats FHB through a coordinated dual mechanism: direct inhibition of the fungus via specific metabolites like Macrolactin A, and simultaneous reprogramming of the host defense and metabolic landscape. These findings provide a scientific foundation for developing this strain as an effective biocontrol agent.

## 1. Introduction

Fusarium head blight (FHB), caused predominantly by the *Fusarium graminearum* species complex, is a devastating fungal disease affecting wheat and other small-grain cereals globally [1,2]. Beyond inflicting substantial yield losses, FHB leads to the accumulation of harmful mycotoxins, such as deoxynivalenol (DON) and zearalenone (ZEN), in grains, severely compromising grain quality and posing a significant threat to food security, livestock health, and human well-being [3,4,5]. The extensive and prolonged application of chemical fungicides, a primary management strategy, has led to the emergence of resistant pathogen strains [6], and some fungicides at sub-lethal concentrations have been shown to stimulate increased mycotoxin production by the fungus [7,8]. Furthermore, the application of chemical fungicides can increase mycotoxin contamination in grain, for instance, by exerting sublethal stress that triggers mycotoxin biosynthesis in pathogens or by selecting for resistant fungal populations that escape control [9]. This underscores the need for alternative strategies, such as biocontrol, which aims to suppress pathogen growth and mycotoxin production directly. These challenges, coupled with the limited availability of wheat varieties that combine high yield, superior quality, and robust FHB resistance [10], underscore the urgent need for sustainable alternatives. Biological control, which utilizes antagonistic microorganisms or their metabolites, has emerged as a highly promising component of integrated disease management due to its inherent advantages of safety and environmental compatibility. Despite its potential, significant challenges in consistent field efficacy and mechanistic understanding impede its widespread implementation [11]. Recent progress continues to advance our knowledge of pathogen biology and control strategies, including biological control [12,13].

Among various biocontrol agents, bacteria from the genera *Bacillus* and *Pseudomonas* are the most extensively studied and representative agents for the biological control of FHB [11,14]. Notably, members of the *Bacillus* genus have garnered significant attention in biocontrol research due to their ability to synthesize a diverse array of antifungal secondary metabolites, such as lipopeptides, polyketides, and bacteriocins, and to form robust, environmentally persistent spores that facilitate industrial-scale production [15,16,17]. *Bacillus* species, particularly those within the *B. velezensis* clade, are renowned as prolific producers of these broad-spectrum antimicrobial compounds. Representative species with demonstrated efficacy against *F. graminearum* include *B. subtilis*, *B. amyloliquefaciens*, and *B. velezensis* [18,19,20,21,22]. For instance, field application of *B. amyloliquefaciens*RC218 significantly reduced the FHB disease index by 58.6% and concurrently decreased grain DON content by 50.3% [22]. Although *Bacillus velezensis* strain 20507 has shown preliminary antagonistic potential, including a demonstrated inhibition rate exceeding 60% against *F. graminearum* in plate assays [23], a comprehensive understanding of its biocontrol efficacy, particularly the tripartite molecular interactions and the specific chemical basis of its activity, is lacking. Crucially, it remains unknown whether strain 20507 possesses a unique metabolic profile or strain-specific antagonistic traits that distinguish it from other *B. velezensis* strains and contribute to a potentially novel mechanism of action against FHB. This gap underscores the need for a systematic investigation to elucidate its full biocontrol potential and originality. In parallel, *Pseudomonas* species, such as *P. fluorescens* and *P. chlororaphis*, also exhibit promising biocontrol potential. Notably, the biocontrol strain *P. chlororaphis*ZJU60, isolated from the wheat head microbiome, achieved 50.0–70.0% control efficacy against FHB in field trials and significantly reduced DON accumulation in grains [24]. The biocontrol mechanism of these agents is often multifaceted, involving not only direct inhibition of pathogen growth but also the induction of plant systemic resistance (ISR) and modulation of host physiological responses, which underscores the complexity of the tripartite (host–pathogen–antagonist) interaction and the need for systematic, multi-faceted investigations.

To deconvolute these intricate interactions, the advent of high-throughput omics technologies, such as transcriptomics and metabolomics, has provided powerful tools [25,26]. Transcriptomics enables the delineation of genome-wide transcriptional reprogramming in both the host and the pathogen, thereby revealing key pathways involved in defense and pathogenesis. Conversely, metabolomics allows for the comprehensive profiling of the chemical arsenal produced by a biocontrol agent, which facilitates the identification of specific bioactive metabolites responsible for the observed phenotypes. An integrated transcriptomic and metabolomic approach thus offers a holistic strategy to bridge the biochemical potential of a biocontrol agent with its functional consequences within a tripartite host–pathogen–antagonist system. To address the aforementioned knowledge gap, we hypothesize that the fermentation broth of *Bacillus velezensis* 20507 suppresses FHB through a dual-pronged mechanism: (i) directly impairing *F. graminearum* growth and physiology by disrupting core metabolic and cellular processes via specific antifungal metabolites, and (ii) indirectly enhancing wheat resistance by priming host defense signaling and reprogramming primary and secondary metabolic pathways. The present study was therefore designed to test these two specific aspects of the hypothesis. To this end, we integrated in vitro and *in planta* efficacy assessments with a dual RNA-seq transcriptomic analysis of both pathogen and host, coupled with LC-MS/MS-based metabolomic profiling of the broth. This multi-omics approach aims to elucidate the comprehensive antagonistic mechanism of *B. velezensis* 20507 fermentation broth and provide a solid foundation for its development as a novel biocontrol agent against FHB.

## 2. Materials and Methods

### 2.1. Fungal Strains and Culture Conditions

The phytopathogenic fungus *F. graminearum* (CGMCC 3.3488) and the biocontrol bacterium *B. velezensis* (CGMCC 20507) used in this study were obtained from the China General Microbiological Culture Collection Center. *F. graminearum* was cultured and activated on Potato Dextrose Agar (PDA) medium at 28 °C for 5 days. For *B. velezensis*, single colonies were inoculated into Luria–Bertani (LB) liquid medium and incubated at 28 °C with shaking at 180 rpm for 24 h to obtain fresh bacterial culture for subsequent fermentation and antagonistic assays.

### 2.2. Dual-Culture Antagonism Test In Vitro

The fermentation broth of *B. velezensis* 20507 was prepared by inoculating a single colony into LB liquid medium, followed by incubation at 28 °C with shaking at 180 rpm for 72 h. The culture was then centrifuged (8000× *g*, 10 min, 4 °C), and the supernatant was filter-sterilized (0.22 μm pore size) to obtain the cell-free fermentation filtrate (CFF) used for bioassays. Antifungal activity of the CFF was evaluated using an agar well diffusion method, a modified version of the standard dual-culture method [27]. Briefly, a 5 mm diameter mycelial plug taken from the actively growing margin of a 3-day-old *F. graminearum* culture was placed at the center of a fresh PDA plate. Four equidistant wells (6 mm in diameter) were then created in the agar using a sterile cork borer, each positioned approximately 3 cm from the central plug. Subsequently, 0.1 mL of the CFF was dispensed into each well. For controls, 0.1 mL of sterile LB medium was added to the wells in control plates, all of which also received the central pathogen plug. All plates were sealed with parafilm and incubated at 25 °C in darkness. Fungal radial growth was measured daily. The antagonistic effect was quantified at 3 and 5 days post-inoculation by comparing the pathogen’s radial growth in treated plates to that in controls. The percentage inhibition of mycelial growth (PIMG) was calculated as: PIMG (%) = [(Rc − Rt)/Rc] × 100, where Rc and Rt represent the radial growth (cm) of the pathogen in the control and treated plates, respectively [28]. The experiment included three biological replicates, each consisting of three technical replicate plates (n = 9). These protocols were adapted from previously published work [27,28].

### 2.3. Time-Course Assessment of Antifungal Metabolite Production

To determine the optimal fermentation duration for the production of antifungal metabolites, a time-course experiment was performed. *B*. *velezensis* 20507 was inoculated into LB liquid medium at an initial density of ~1 × 10^8^ CFU/mL and incubated at 28 °C with shaking at 180 rpm. Samples of the culture were collected daily from day 1 to day 7. Each sample was centrifuged (8000× *g*, 10 min, 4 °C) and the supernatant was filter-sterilized (0.22 μm pore size) to obtain the cell-free fermentation broth. The antifungal activity of each daily broth was assessed using a modified agar well diffusion method. Briefly, a 5 mm mycelial plug of *F. graminearum* was placed at the center of a fresh PDA plate. Then, 0.1 mL of the filter-sterilized broth from a specific fermentation day was applied directly onto the agar surface at a single point adjacent to the fungal plug. A control plate received 0.1 mL of sterile LB medium. The radial growth of the fungus was measured after 5 days of incubation at 25 °C in darkness, and the percentage inhibition of mycelial growth (PIMG) was calculated as described in Section 2.2. The experiment included three biological replicates. The antifungal activity of the fermentation broth against *F. graminearum* increased over the fermentation period, reaching a plateau with an inhibition rate of approximately 75% by day 5; no significant further increase was observed on days 6 and 7 (Figure S1). Therefore, the 5-day fermentation broth was selected for and used in all subsequent plant inoculation experiments (treatments Bv and BvFg).

### 2.4. Plant Inoculation Assay and Sample Collection for Transcriptome Analysis

Plant growth and experimental design. Seeds of the wheat cultivar (*Triticum aestivum* L. cv. Long mai 35) were sown in sterile humus substrate, with 20 seeds per pot (12 pots in total). Plants were maintained in a growth chamber at 28 °C under a 16/8 h (light/dark) photoperiod and 70% relative humidity for 23 days until the seedling stage. The experiment comprised four treatments: (1) mock-inoculated control (CK, wounded but not inoculated); (2) *F. graminearum* inoculation (Fg); (3) *Bacillus velezensis* 20507 fermentation broth application (Bv); and (4) co-inoculation of *F. graminearum* and *B. velezensis* 20507 fermentation broth (BvFg). For the Fg and BvFg treatments, the fungal inoculum consisted of one compact mycelial pellet (approximately 0.6 cm in diameter). This form and amount of inoculum were selected to ensure consistent, localized infection at the wound site, which is critical for synchronized disease development and precise sampling for transcriptome analysis. For the BvFg co-inoculation treatment, 20 µL of the bacterial fermentation broth was applied directly into the wound first, followed immediately by placing one fungal mycelial pellet into the same wound. This sequential application was designed to test the protective effect of the broth against subsequent fungal challenge. Each treatment included three biological replicates, with each replicate consisting of three individual seedling stems (nine stems per treatment in total).

Preparation of inoculate. *F. graminearum* was pre-cultured on potato dextrose agar (PDA) for 7 days. Mycelial pieces (approximately 0.2 × 0.2 cm) free of agar were aseptically excised from the colony margins and transferred into 300 mL of potato dextrose broth (PDB). The culture was incubated at 28 °C with shaking at 180 rpm for 72 h to obtain compact mycelial pellets (ca. 0.6 cm in diameter). Pellets were harvested by centrifugation at 5000× *g* for 10 min and washed twice with sterile saline. The fermentation broth of *B. velezensis* 20507 was prepared as described in Section 2.2.

Seedling inoculation. To facilitate infection and enable standardized sampling of the interaction site for transcriptomics, a controlled wound inoculation assay was employed. Prior to plant inoculation, the *B. velezensis* 20507 fermentation broth was concentrated 10-fold using a rotary evaporator at 50 °C. The potent antifungal activity of this concentrated broth was confirmed and quantitatively validated in vitro using a modified agar well diffusion method, which demonstrated a consistent growth inhibition rate exceeding 75% against *F. graminearum*. A longitudinal wound (ca. 1 cm long, depth < 50% of stem diameter) was made on the lowest internode of each seedling using a sterile scalpel. According to the treatment, the wound was inoculated with one *F. graminearum* pellet, 20 µL of the concentrated *B. velezensis* 20507 fermentation broth, or both. For the co-inoculation treatment (BvFg), the concentrated fermentation broth was applied first, followed immediately by the fungal pellet placed into the same wound. Control seedlings (CK) were wounded but received no inoculum. While this method does not replicate natural floral infection, it provides a robust system for synchronizing infection and analyzing localized plant–pathogen–biocontrol interactions in a defined tissue.

Disease assessment and sample collection. After inoculation, the treated stem segments were placed on moist filter paper in Petri dishes to maintain humidity and promote symptom development. Disease progression was monitored daily through visual observation for the development and expansion of symptoms, including water-soaking, tissue browning, and lesion formation. At 5 days post-inoculation, when distinct lesions were consistently visible in the Fg-treated plants, a 2 cm stem segment centered precisely on the inoculation site was excised from each seedling. For each biological replicate, segments from three seedlings were pooled, immediately frozen in liquid nitrogen, and stored at −80 °C for subsequent RNA extraction. The entire experiment was performed with three independent biological replicates. The total RNA extracted from these stem segments contained transcripts from both wheat and the infecting fungus. Subsequently, a dual RNA-seq bioinformatics approach was employed to analyze the transcriptional profiles of the host and the pathogen from this mixed RNA.

### 2.5. RNA Extraction, Library Construction, and Sequencing

Total RNA was extracted from approximately 100 mg of frozen wheat stem tissue per sample using the RNA prep Pure Plant Kit (Tiangen Biotech, Beijing, China) according to the manufacturer’s protocol. RNA concentration and purity were measured with a Nano Drop spectrophotometer (Thermo Fisher Scientific, Waltham, MA, USA) and a Qubit 2.0 Fluorometer (Thermo Fisher Scientific, Waltham, MA, USA), respectively. RNA integrity was evaluated on an Agilent 2100 Bioanalyzer (Agilent Technologies, Santa Clara, CA, USA). Only RNA samples meeting the following criteria were used for subsequent library construction: OD260/280 ratio 1.8–2.2, OD260/230 ratio > 2.0, and RNA integrity number (RIN) ≥ 7.0. The transcriptomic data derived from this RNA reflect the *in planta* response of wheat to both pathogen challenge and biocontrol treatment.

RNA sequencing libraries were constructed and sequenced by Biomarker Technologies Co., Ltd (Beijing, China). Briefly, mRNA was enriched from total RNA using oligo(dT) magnetic beads and then fragmented. First-strand cDNA synthesis was performed with random hexamer primers, followed by second-strand synthesis. The resulting double-stranded cDNA was purified with AM Pure XP beads, end-repaired, A-tailed, and ligated to Illumina adapters. Fragments of 300–400 bp were size-selected and amplified by PCR to generate sequencing-ready libraries. Library quality was assessed on an Agilent 2100 Bioanalyzer (Agilent Technologies, Santa Clara, CA, USA), and concentration was quantified using a Qubit 2.0 Fluorometer and qPCR. Libraries with concentrations above 2 nM were subjected to paired-end 150 bp (PE150) sequencing on an Illumina Nova Seq 6000 platform (Illumina, San Diego, CA, USA).

### 2.6. Bioinformatics Analysis of RNA-Seq Data

Data preprocessing and alignment. Raw paired-end reads were processed with fastp (v0.20.0) [24] to remove adapter sequences, poly-N regions, and low-quality bases, yielding high-quality clean reads. The clean reads were aligned separately to the reference genomes of *Triticum aestivum* (IWGSC Ref Seq v2.1) and *F. graminearum* (strain PH-1) using HISAT2 (v2.2.1) [29].

Transcript quantification and normalization. Transcript abundances were estimated in a reference-guided manner with StringTie (v2.2.1) [30,31]. Gene expression levels were normalized as FPKM (Fragments Per Kilobase of transcript per Million fragments mapped) to account for differences in transcript length and sequencing depth.

Differential expression analysis. Read counts per gene were used as input for DESeq2 (v1.30.1) [32] to identify differentially expressed genes (DEGs) between the designated comparisons. Genes with an adjusted *p*-value (FDR) < 0.05 and |log_2_ (fold change) | > 1 were considered significantly differentially expressed. For all comparisons reported in this study, the direction of regulation is defined relative to the first-named group. In a comparison labeled “A vs. B”, genes reported as “up-regulated” have significantly higher expression in group A relative to group B, and genes reported as “down-regulated” have significantly lower expression in group A relative to group B.

Functional enrichment analysis. Gene Ontology (GO) and Kyoto Encyclopedia of Genes and Genomes (KEGG) pathway enrichment analyses of the DEGs were performed using the cluster Profiler package (v4.0.5) [33,34]. Terms with a corrected *q*-value < 0.05 were regarded as significantly enriched.

### 2.7. Quantitative Real-Time PCR (qRT-PCR) Validation

To validate the RNA-seq results, qRT-PCR was performed on 20 differentially expressed genes (DEGs)—10 from *F. graminearum*, and 10 from *T. aestivum*. The same RNA samples used for RNA-seq were reverse-transcribed into cDNA using the PrimeScript RT reagent Kit with gDNA Eraser (TaKaRa, Japan). qRT-PCR was conducted on a QuantStudio 5 system (Applied Biosystems, USA) with TB Green Premix Ex Taq II (TaKaRa, Japan). Each 20 µL reaction contained 10 µL of TB Green Premix, 0.8 µL each of forward and reverse primers (10 µM), 2 µL of cDNA, and 6.4 µL of nuclease-free water. The thermal profile was: 95 °C for 30 s; 40 cycles of 95 °C for 5 s and 60 °C for 30 s. Melt curve analysis confirmed amplification specificity. All reactions were run in three technical replicates per biological replicate. Primer sequences are listed in Table S1. The reference genes used for normalization were *actin* for *T. aestivum* and *β-tubulin* for *F. graminearum*. Relative expression was calculated using the 2^−ΔΔCt^ method [35].

### 2.8. Isolation and Identification of Antifungal Metabolites from the Fermentation Broth

To comprehensively characterize the antifungal compounds responsible for the observed biocontrol activity, we performed a sequential chemical analysis that integrated untargeted metabolomic profiling, bioassay-guided fractionation, and targeted characterization. First, the fermentation broth of *Bacillus velezensis* 20507 was concentrated 10-fold using a rotary evaporator. Bioactive components were first isolated by chromatographic fractionation using a ClearFirst-3000Max protein purification system (Shanghai Shanpu Technology Co., Ltd., Shanghai, China) equipped with a DEAE Seplife HP column (Sunresin Technology Co., Ltd., Xi’an, Shaanxi, China; 10 μm, 0.7 × 2.5 cm; column stored at 4–8 °C). The mobile phase consisted of 0.05 mol/L Tris buffer (pH 8.3, phase A) and 0.05 mol/L Tris buffer containing 2 mol/L NaCl (pH 8.3, phase B), delivered at a flow rate of 1 mL/min. Fractions were collected according to chromatographic peaks, concentrated under vacuum, reconstituted in sterile water, and screened for antifungal activity against *F. graminearum* using the agar well diffusion method described in Section 2.2. Two fractions that consistently exhibited strong inhibition—designated Fr A (retention time 12–14 min) and Fr B (retention time 18–20 min)—were selected for targeted chemical identification. These Fr A and Fr B were subsequently subjected to untargeted LC-MS/MS analysis on an UltiMate 3000 UHPLC system (Thermo Fisher Scientific, Waltham, MA, USA) coupled to a Q Ex active mass spectrometer (Thermo Fisher Scientific, Waltham, MA, USA) for compound identification. Chromatographic separation was performed on a Thermo Hypersil Gold C18 column (Thermo Fisher Scientific, Waltham, MA, USA; 1.9 μm, 2.1 × 100 mm) maintained at 40 °C, with a mobile phase of 0.1% (*v*/*v*) formic acid in water (A) and methanol (B). The gradient program was: 0–1 min, 10% B; 1–10 min, 10–90% B; 10–12 min, 90% B; 12.1–15 min, 10% B. Mass detection was carried out in positive and negative ion modes using a heated electrospray ionization source (source temperature 310 °C, capillary temperature 320 °C, sheath gas 30 arb, auxiliary gas 10 arb, spray voltage 3.0 kV/2.8 kV). Full-scan data (*m*/*z* 100–1500, resolution 70,000) and data-dependent MS/MS scans (top 10 ions, stepped collision energies of 10, 28, and 35 eV) were acquired. Raw data were processed with Compound Discoverer (v3.2, Thermo Fisher Scientific, Waltham, MA, USA) and searched against multiple databases (ChemSpider, CHEBI, ChEMBL, mzCloud) for preliminary annotation. The raw mass spectrometry data and processed peak tables from the untargeted metabolomics analysis have been deposited in the Zenodo repository under accession DOI: https://doi.org/10.5281/zenodo.19279834. The confidence of metabolite identification was assigned according to the Metabolomics Standards Initiative (MSI) levels: Level 1: Confidently identified compounds, matched by retention time and MS/MS spectrum to an authentic standard analyzed under identical conditions. Level 2: Putatively annotated compounds, based on diagnostic MS/MS spectral similarity to a reference spectrum in a public or commercial library. Level 3: Putatively characterized compound classes, based on physicochemical properties or spectral similarity to compounds of a known class. Level 4: Unknown compounds. In this study, compounds were annotated primarily at MSI Level 2 based on matching accurate mass and MS/MS fragmentation patterns against spectral libraries (e.g., mzCloud). The complete list of annotated features, including their tentative identification, MSI confidence level, and relative abundance, is provided in Supplementary Table S2.

## 3. Results

### 3.1. B. velezensis 20507 Fermentation Broth Inhibits F. graminearum Mycelial Growth In Vitro

**Figure 1:**
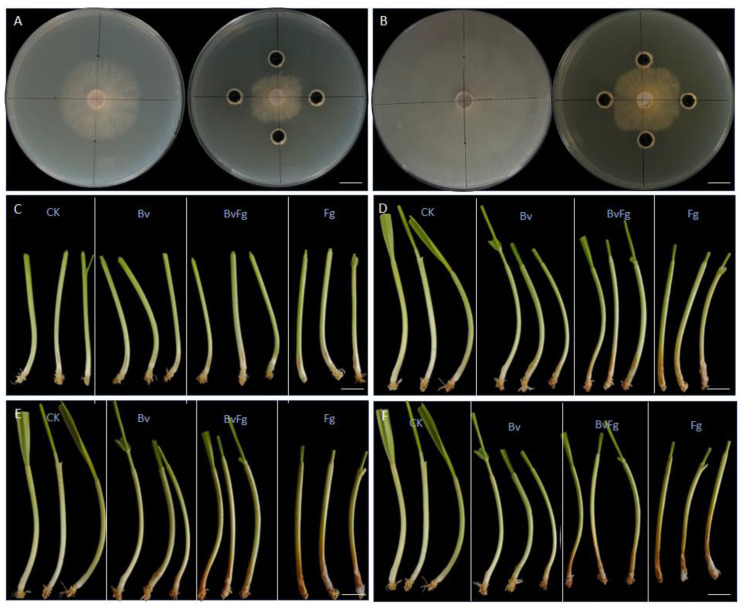
In vitro and in vivo antagonistic activity of *Bacillus velezensis* 20507 fermentation broth against *Fusarium graminearum*. (**A**,**B**) In vitro antagonism assay using the agar well diffusion method. Representative images of the assay at 3- and 5-days post-inoculation (dpi), respectively. In each panel, the left plate shows the control group inoculated with a single agar plug of *F. graminearum* (Fg) at the center, while the right plate shows the treatment group inoculated with *F. graminearum* at the center and 0.1 mL of *B. velezensis* 20507 fermentation broth applied at each of the four corners. Scale bars: 2.0 cm. (**C**–**F**) Biocontrol effect on wheat seedlings. Disease symptoms on trimmed wheat stem segments at 1, 3, 4, and 5 days post-inoculation (dpi), respectively. The treatments are as follows: CK, mock-inoculated control (wounded but not inoculated with pathogen); Fg, inoculated with *F. graminearum* alone; Bv, inoculated with *B. velezensis* 20507 fermentation broth; BvFg, co-inoculated with *F. graminearum* and *B. velezensis* 20507 fermentation broth. Prior to inoculation and imaging, leaves and roots were trimmed to standardize the stem segments and facilitate clear visualization of lesion development on the stem. Scale bars: 1.0 cm.

The in vitro antagonistic activity of *Bacillus velezensis* 20507 fermentation broth against *Fusarium graminearum*, the causal agent of Fusarium head blight (FHB), was evaluated using an agar well diffusion method. As shown in Figure 1, the broth exhibited significant inhibition of fungal mycelial growth. In control plates, a single agar plug of *F. graminearum* grew radially, forming a dense, uniform colony that nearly covered the entire plate by 5 days post-inoculation (dpi) (Figure 1A,B; left panels). In contrast, when 0.1 mL of fermentation broth was applied at four points surrounding the central fungal plug, mycelial growth was markedly suppressed. A distinct inhibition zone formed between the application points and the fungal colony, preventing the colony from reaching the plate edges even at 5 dpi (Figure 1A,B; right panels). Based on radial growth measurements, the percentage inhibition of mycelial growth (PIMG) was calculated as 59.8%. These results demonstrate the potent, direct antifungal activity of *B. velezensis* 20507 fermentation broth against *F. graminearum* in vitro, supporting its potential as a biocontrol agent for FHB management.

### 3.2. Fermentation Broth of B. velezensis 20507 Alleviates Disease Symptoms in Wheat Seedlings

To evaluate the protective effect of *B. velezensis* 20507 fermentation broth against Fusarium head blight *in planta*, a seedling inoculation assay was conducted. Wheat stems were observed for disease progression from 1 to 5 days post-inoculation (dpi) across four treatments: mock-inoculated control (CK), pathogen-only (F. graminearum, Fg), fermentation broth-only (Bv), and co-inoculation (BvFg) (Figure 1C–F). In the Fg treatment, typical FHB symptoms, including water-soaked lesions and tissue browning, rapidly developed and expanded along the stems over time. By 5 dpi, severe necrosis was evident (Figure 1C–F, Fg). In contrast, stems in the CK and Bv treatments remained healthy throughout the observation period, with no visible lesions, indicating that the fermentation broth itself was not phytotoxic (Figure 1C–F, CK and Bv). Notably, in the BvFg co-inoculation treatment, disease development was significantly suppressed compared to the Fg treatment. While mild symptoms occasionally appeared, the lesion expansion was markedly inhibited, and the stems largely maintained a healthy appearance similar to the controls (Figure 1C–F, BvFg). These results visually demonstrate that the application of *B. velezensis* 20507 fermentation broth can effectively reduce disease severity and alleviate the symptoms caused by *F. graminearum* infection in wheat seedlings, confirming its biocontrol efficacy *in planta*.

### 3.3. Antifungal Activity of B. velezensis 20507 Fermentation Broth Is Time-Dependent

**Figure 2:**
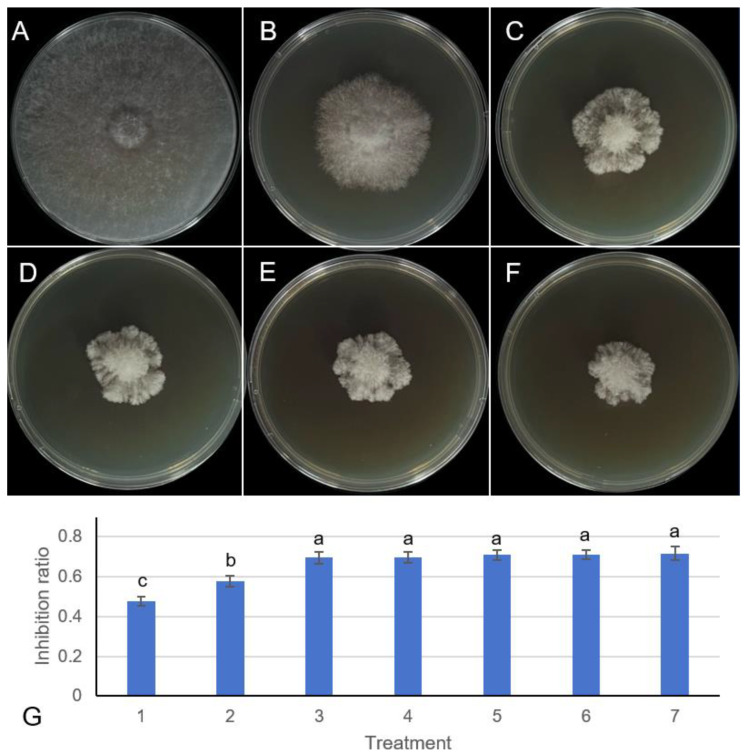
Effect of fermentation time on the antifungal activity of *Bacillus velezensis* 20507 fermentation broth against *Fusarium graminearum* using an agar well diffusion method. (**A**) Growth of *F. graminearum* on a potato dextrose agar (PDA) plate (control) at 5 days post-inoculation. (**B**–**F**) Growth of *F. graminearum* on PDA plates at 5 days post-inoculation, showing the antifungal activity of 0.1 mL *B. velezensis* 20507 fermentation broth harvested after 1, 2, 3, 4, and 5 days of fermentation, respectively. The broth was added to the center of each plate. (**G**) Inhibition rates of *F. graminearum* by the fermentation broth collected from day 1 to day 7 of fermentation. Data are presented as mean ± standard deviation (n = 3). Different lowercase letters above the bars indicate statistically significant differences among the treatments as determined by one-way ANOVA followed by Duncan’s multiple range test (*p* < 0.05). Scale bars: 2.0 cm (**A**–**F**).

The production of antifungal metabolites by *Bacillus velezensis* 20507 was found to be strongly dependent on fermentation duration. As shown in Figure 2, the antifungal efficacy of the fermentation broth against *F. graminearum* was assessed daily. In the control plate, *F. graminearum* grew unrestrictedly, forming a dense colony (Figure 2A). Broth harvested after 1 day of fermentation exhibited only minimal inhibitory activity (Figure 2B), corresponding to a low inhibition rate in the quantitative analysis (Figure 2G). Antifungal activity increased markedly from day 2, as evidenced by the appearance of a clear inhibition zone (Figure 2C). The activity peaked and was sustained in broths collected from days 3 to 7, which produced the largest and most distinct inhibition zones (Figure 2D–F, representative images from day 3–5 are shown), completely suppressing fungal growth at the application site. The quantitative inhibition rates during this period reached a plateau, with a maximum of approximately 75% and no statistically significant differences observed between days 3 and 7 (Figure 2G). This time-course study demonstrates that a fermentation period of 3 to 7 days is optimal for *B. velezensis* 20507 to produce highly effective antifungal compounds against *F. graminearum*, providing important guidance for the efficient production of this biocontrol agent.

### 3.4. RNA Sequencing and Mapping Efficiency

Based on the sequencing statistics provided in Table S3, high-quality RNA-seq data were successfully generated from all twelve samples, encompassing the four treatment groups (CK, Fg, Bv, BvFg) with three biological replicates each. The total number of raw reads per library ranged from approximately 52.1 to 56.3 million, yielding a total base count between 1.56 × 10^10^ and 1.68 × 10^10^ per sample. The data exhibited uniformly high quality, as indicated by the consistently low percentage of ambiguous bases (N ≤ 0.02%), an average GC content of 53.2%, and high sequencing accuracy with Q20 and Q30 scores averaging ≥ 98.7% and ≥95.0%, respectively. These metrics confirm the robustness and depth of the sequencing data suitable for subsequent transcriptomic analyses.

Based on the data presented in Table S4, the mapping efficiency of sequencing reads to the *F*. *graminearum* genome revealed a dramatic, treatment-specific pattern. In samples inoculated with the pathogen alone (Fg), an average of 7.5% of the total reads uniquely mapped to the fungal genome. In stark contrast, samples that were co-inoculated with the pathogen and the *B. velezensis* 20507 fermentation broth (BvFg) showed a drastic reduction in this metric, with an average unique mapping rate of only 0.11%, representing an approximate 68-fold decrease. This substantial reduction in mappable fungal transcripts provides direct sequencing-level evidence for the potent suppressive effect of the bacterial broth on the pathogen’s transcriptional activity *in planta*. The uniquely mapped reads were approximately evenly distributed between genomic strands, and the proportion of multiple-mapped reads was negligible (<0.05%), confirming the high specificity of the alignment.

Based on the data presented in Table S5, the efficiency of aligning RNA-seq reads to the *Triticum aestivum* (wheat) genome was consistently high across most treatments, with a notable exception. The average unique read mapping rates for the control (CK), *B. velezensis* 20507 broth application (Bv), and co-inoculation (BvFg) groups were 88.3%, 86.1%, and 87.5%, respectively, indicating robust capture of the wheat transcriptome. In stark contrast, samples subjected to *F*. *graminearum* inoculation alone (Fg) exhibited a significant and consistent reduction in mapping efficiency, with an average unique mapping rate of 76.2%. The marked decrease in the proportion of reads uniquely mapping to the wheat genome in the Fg treatment (76.2% vs. 86.1–88.3% in other groups) is a direct sequencing-level observation. In the context of this interaction transcriptomics experiment, a likely primary explanation for this reduced host mapping rate is the substantial increase in *F. graminearum* biomass and, consequently, the relative abundance of fungal RNA within the total RNA pool extracted from the infected tissue. This would naturally reduce the proportional contribution of wheat-derived reads available for alignment to its own genome. While a pathogen-induced suppression of host transcription may also be a contributing factor, the pronounced shift in RNA composition provides a robust, quantifiable signature of successful pathogen colonization and proliferation in the Fg samples, which aligns with the phenotypic disease symptoms observed. This dataset provides a clear baseline of the pathogen-dominated transcriptomic state, against which the counteracting effects of the biocontrol broth (BvFg) can be effectively compared.

### 3.5. B. velezensis 20507 Fermentation Broth Directly Inhibits Pathogen Growth and Disrupts Its Core Metabolism

**Figure 3:**
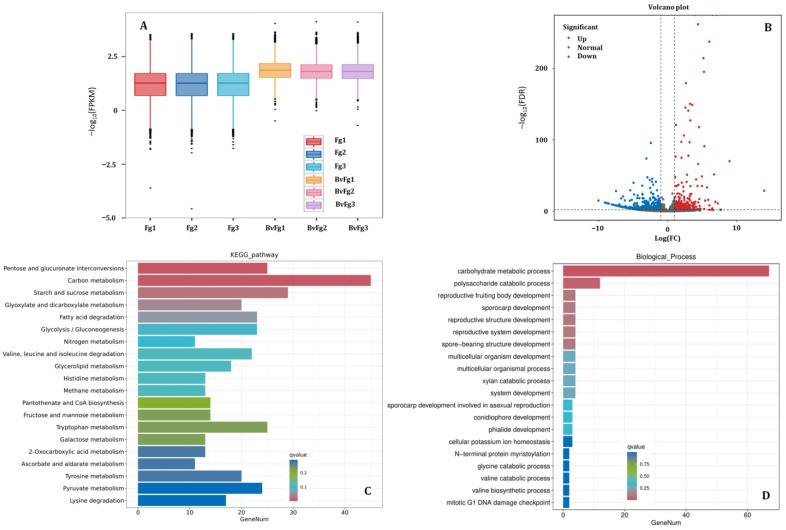
Comparative transcriptomic analysis of *F. graminearum* in response to *Bacillus velezensis* 20507 fermentation broth. (**A**) Box plots showing the distribution of FPKM values for all *F. graminearum* genes under different treatments. The y-axis is plotted on a −log_10_ scale. (**B**) Volcano plot of differentially expressed genes (DEGs) in *F. graminearum* from the comparison between plants inoculated with *F. graminearum* alone (Fg) and those co-inoculated with *F. graminearum* and *B. velezensis* 20507 broth (BvFg). (**C**) KEGG pathway and (**D**) Gene Ontology (Biological Process) enrichment analyses of the *F. graminearum* DEGs identified in the Fg vs. BvFg comparison. Treatments: CK, mock-inoculated control; Bv, inoculated with *B. velezensis* 20507 fermentation broth; Fg, inoculated with *F. graminearum* alone; BvFg, co-inoculated with *F. graminearum* and the fermentation broth. All RNA-seq reads from Fg and BvFg samples were uniquely mapped to the *F. graminearum* reference genome.

Global gene expression patterns in *F. graminearum* across all samples are presented in Figure 3A as box plots of FPKM (Fragments Per Kilobase of transcript per Million mapped reads) values. The data are plotted on a −log10 scale to better visualize the distribution. As shown, the median FPKM values (plotted as higher −log10 (FPKM) positions) for the BvFg group are substantially lower than those for the Fg group. This indicates that the overall transcriptional output of *F. graminearum* was significantly suppressed when co-inoculated with the *B. velezensis* fermentation broth compared to the pathogen-only condition.

Subsequent analysis identified 1510 differentially expressed genes (DEGs) (Figure 3B), with a strong bias towards down-regulation (1192 down-regulated vs. 318 up-regulated), reinforcing the trend of widespread transcriptional suppression induced by the fermentation broth. Functional enrichment analysis of these DEGs linked this suppression to the disruption of core metabolic pathways. Gene Ontology (GO) enrichment revealed that the most significantly affected biological processes were “carbohydrate metabolic process” (GO:0005975) and “polysaccharide catabolic process” (GO:0000272) (Figure 3C), pointing to interference with energy metabolism and cell wall integrity. This finding was corroborated by KEGG pathway analysis, which showed significant enrichment in key carbohydrate metabolism pathways, including “Pentose and glucuronate interconversions” (ko00040), “Carbon metabolism” (ko01200), “Starch and sucrose metabolism” (ko00500), and “Glyoxylate and dicarboxylate metabolism” (ko00630) (Figure 3D). The concerted down-regulation of genes within these essential pathways suggests that the fermentation broth of *B. velezensis* 20507 likely impairs the pathogen’s energy production, carbon utilization, and cell wall maintenance, thereby mechanistically contributing to its growth inhibition and the overall biocontrol efficacy.

### 3.6. Global Transcriptomic Alterations in Wheat Reveal Pathogen-Induced Suppression and Biocontrol Agent-Mediated Modulation

**Figure 4:**
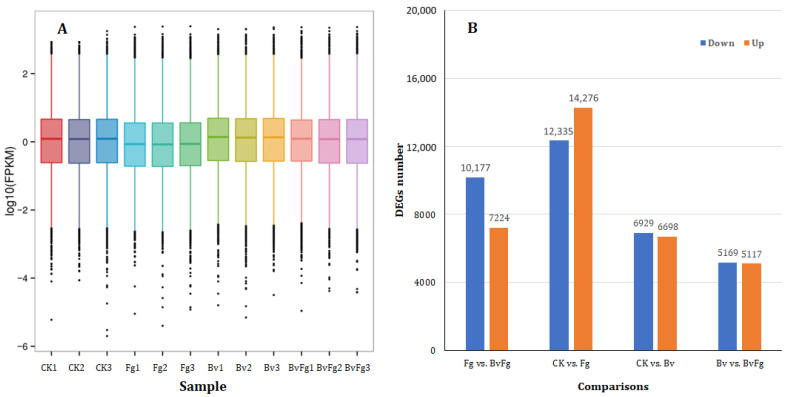
Transcriptomic profiling of wheat (*Triticum aestivum*) in response to *Fusarium graminearum* and *Bacillus velezensis* 20507 fermentation broth. (**A**) Box plots showing the distribution of FPKM values for all wheat genes under different treatments. The *y*-axis is plotted on a log10 scale. The lower median position for the Fg group reflects a lower median FPKM, indicating a global reduction in host transcript abundance induced by the pathogen. (**B**) Differentially expressed genes (DEGs) identified from the four pairwise comparisons: CK vs. Bv, CK vs. Fg, Bv vs. BvFg, and Fg vs. BvFg. Treatments: CK, mock-inoculated control; Fg, inoculated with *F. graminearum* alone; Bv, inoculated with *B. velezensis* 20507 fermentation broth; BvFg, co-inoculated with *F. graminearum* and the fermentation broth. All RNA-seq reads were uniquely mapped to the *Triticum aestivum* reference genome for this analysis.

Transcriptomic profiling of wheat stems under different treatments revealed distinct global gene expression patterns (Figure 4A), shown as box plots of FPKM values on a log10 scale. The median FPKM value (plotted as a lower log10 (FPKM) position) for the Fg group was notably lower than those for the CK, Bv, and BvFg groups. This indicates that pathogen infection alone was associated with a significant reduction in overall host transcript abundance, whereas the application of the fermentation broth, either alone or combined with the pathogen, was associated with the maintenance of higher transcriptional levels.

Differential gene expression analysis confirmed extensive transcriptional reprogramming in wheat stems across all key treatment comparisons (Figure 4B; see also Tables S6–S9 for DEG counts per comparison). The scale of these changes demonstrates that *F. graminearum* infection induced the most pronounced alterations, while the application of *B. velezensis* 20507 fermentation broth, both alone and in combination with the pathogen, also triggered substantial and specific transcriptional shifts in the host. The detailed functional consequences of these reprogramming events—for both up- and down-regulated genes in each critical comparison—are explored in the subsequent KEGG pathway enrichment analyses.

### 3.7. Transcriptional and Metabolic Reprogramming in Wheat in Response to Pathogen Infection and B. velezensis 20507 Biocounteraction

**Figure 5:**
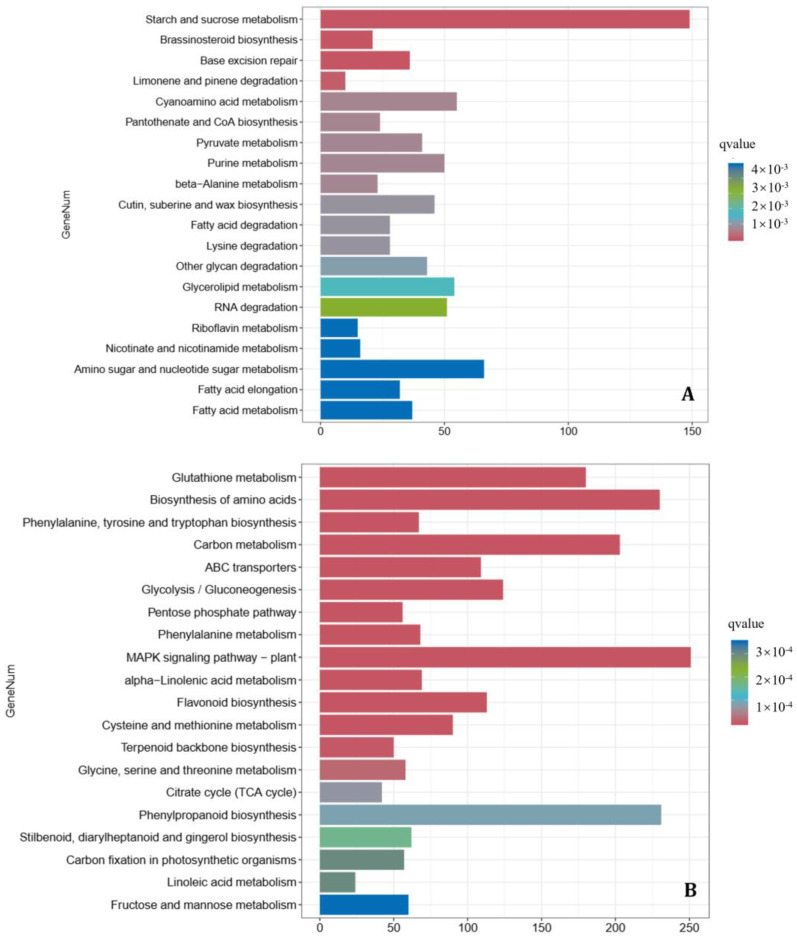
Kyoto Encyclopedia of Genes and Genomes (KEGG) pathway enrichment analysis of the wheat transcriptome in response to different treatments. (**A**) Top enriched KEGG pathways for up-regulated differentially expressed genes (DEGs) in the comparison between plants inoculated with *Fusarium graminearum* alone (Fg) and those co-inoculated with *F. graminearum* and *Bacillus velezensis* 20507 fermentation broth (BvFg). (**B**) Top enriched KEGG pathways for down-regulated DEGs in the Fg vs. BvFg comparison. All RNA-seq reads from wheat samples were uniquely mapped to the *Triticum aestivum* reference genome.

The comparison between pathogen-only and co-inoculation treatments (Fg vs. BvFg) yielded 17,401 differentially expressed genes (DEGs), with a marked bias toward down-regulation (10,177 down-regulated vs. 7224 up-regulated; Table S6). To elucidate the functional mechanisms behind this broad transcriptional counteraction, a KEGG pathway enrichment analysis was performed on these DEGs with separate evaluations for up- and down-regulated sets (Figure 5, Table S6). This refined analysis revealed a distinct, direction-specific reprogramming. The up-regulated DEGs were significantly enriched in starch and sucrose metabolism (ko00500), brassinosteroid biosynthesis (ko00905), base excision repair (ko03410), and monoterpenoid degradation (ko00903) (Figure 5A). This enrichment pattern suggests that the bacterial fermentation broth specifically primes the plant for defense by reallocating energy resources, activating phytohormone signaling, enhancing genome stability maintenance, and potentially modulating signaling or detoxification processes. Conversely, the down-regulated DEGs were predominantly enriched in various primary metabolic pathways, with the most prominent being glutathione metabolism (ko00480), amino acid biosynthesis (ko01230), phenylalanine, tyrosine and tryptophan biosynthesis (ko00400), carbon metabolism (ko01200), and ABC transporters (ko02010) (Figure 5B). This coordinated down-regulation likely represents a strategic metabolic reprogramming, where resources are diverted away from primary growth and biosynthetic processes to fuel the activated defense responses highlighted by the up-regulated gene set. Collectively, these transcriptional reprogramming profiles demonstrate that the bacterial fermentation broth orchestrates a two-pronged host response: it specifically up-regulates key pathways associated with defense priming and stress resilience (e.g., energy reallocation, brassinosteroid signaling, and DNA repair), while concurrently down-regulating fundamental metabolic and biosynthetic processes. This divert-and-channel strategy strongly suggests a resource reallocation mechanism, whereby the host’s metabolic budget is shifted from growth-oriented activities toward sustaining the activated defense program, thereby establishing a coordinated and potentially cost-efficient defense strategy against *F. graminearum* infection.

**Figure 6:**
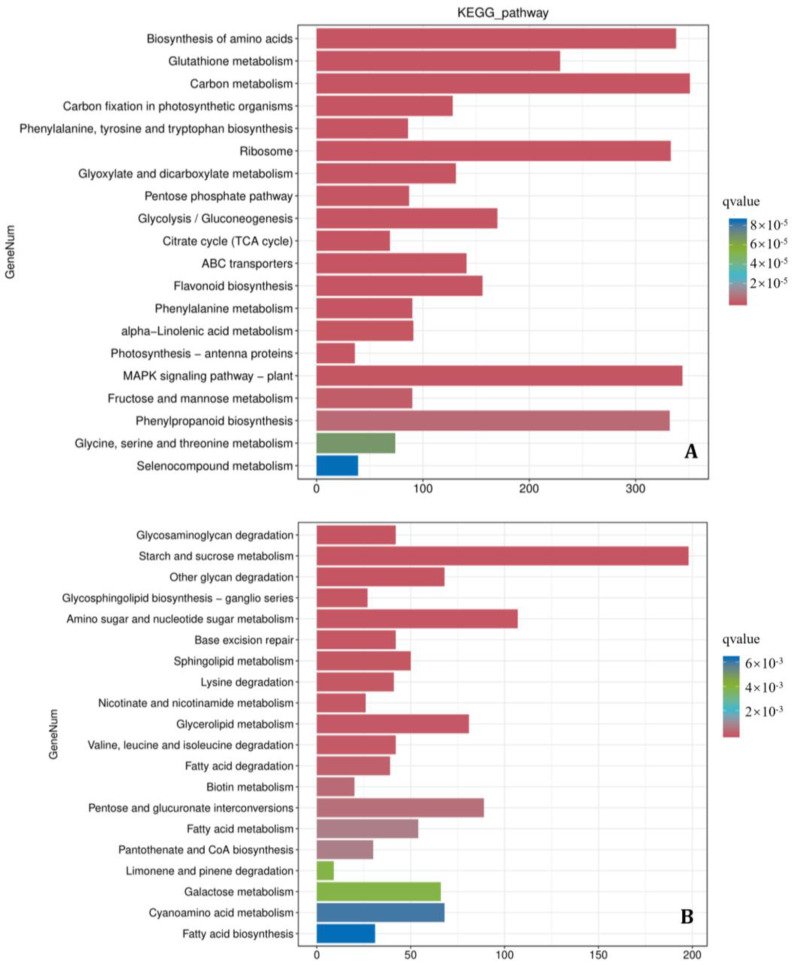
Kyoto Encyclopedia of Genes and Genomes (KEGG) pathway enrichment analysis reveals the impact of *Fusarium graminearum* infection on the wheat transcriptome. (**A**) Top enriched KEGG pathways for up-regulated differentially expressed genes (DEGs) in wheat plants inoculated with *F. graminearum* (Fg) compared to the mock-inoculated control (CK). (**B**) Top enriched KEGG pathways for down-regulated DEGs in the Fg vs. CK comparison. All RNA-seq reads from wheat samples were uniquely mapped to the *Triticum aestivum* reference genome.

Pathogen infection alone (CK vs. Fg) induced the most pronounced transcriptional changes, yielding 26,611 differentially expressed genes (DEGs; 12,335 up-regulated and 14,276 down-regulated; Figure 4B; Table S7). Directional KEGG enrichment analysis of these DEGs (Figure 6, Table S7) revealed a profound yet dichotomous reprogramming of the host transcriptome. The up-regulated DEGs were significantly enriched in defense and stress-related pathways, most notably the MAPK signaling pathway—plant (ko04016), phenylpropanoid biosynthesis (ko00940), and glutathione metabolism (ko00480) (Figure 6A)—indicating an active, induced host defense response. In stark contrast, the down-regulated DEGs were predominantly enriched in fundamental biosynthetic and energy metabolism pathways, including glycosaminoglycan degradation (ko00531), starch and sucrose metabolism (ko00500), other glycan degradation (ko00511), glycosphingolipid biosynthesis (e.g., ko00603), aminosugar and nucleotide sugar metabolism (ko00520), and base excision repair (ko03410). This pattern indicates that *F. graminearum* infection triggers a profound dysregulation of host metabolism. It not only elicits expected defense responses but also instigates a widespread suppression of fundamental biosynthetic, energy, and cellular maintenance pathways. This concerted reprogramming likely reflects a pathogen-induced disruption of core host physiology, compromising metabolic homeostasis and cellular integrity, which collectively contributes to disease susceptibility and pathogen virulence.

Collectively, the comparative transcriptomic analyses delineate a clear antagonistic interplay between the pathogen and the biocontrol agent. Infection by *F. graminearum* alone triggered a dichotomous host response, characterized by the activation of defense pathways (e.g., MAPK signaling, phenylpropanoid biosynthesis) concurrent with a broad suppression of fundamental metabolic and biosynthetic processes (Figure 6). In contrast, the application of the *B. velezensis* 20507 fermentation broth during co-inoculation effectively counteracted this pathogenic reprogramming. The biocontrol agent did not simply reverse the pathogen-induced changes but rather refined and redirected the host response. It potentiated a stronger and more specific up-regulation of core defense and detoxification pathways (e.g., phenylpropanoid biosynthesis, MAPK signaling, glutathione metabolism) that were also induced by the pathogen alone (Figure 5A). Simultaneously, it instigated a distinct down-regulation of primary metabolic pathways—particularly those related to carbohydrate and energy metabolism (e.g., starch and sucrose metabolism)—which differed from the pattern of metabolic suppression caused by the pathogen alone (Figure 5B). This transcriptional reconfiguration suggests a strategic metabolic rebalancing. Therefore, the biocontrol efficacy of *B. velezensis* 20507 can be attributed to its ability to reprogram the host from a state of pathogenic stress and metabolic dysregulation towards a reconfigured, defense-primed state. This reprogramming optimizes resource allocation to sustained defense, thereby enhancing the plant’s resistance against *F. graminearum* infection.

### 3.8. Molecular Basis of Defense Priming and Amplified Response by B. velezensis 20507

Transcriptional reprogramming upon broth application alone (CK vs. Bv). The application of *B. velezensis* 20507 fermentation broth alone triggered substantial transcriptional alterations, as evidenced by 13,627 differentially expressed genes (DEGs; 6929 up-regulated and 6698 down-regulated; Figure 4B; Table S8). Directional KEGG enrichment analysis of these DEGs (Figure S2, Table S8) revealed a targeted reprogramming indicative of defense priming. The up-regulated DEGs were prominently enriched in well-established defense and biosynthesis pathways, most notably phenylpropanoid biosynthesis (ko00940), ABC transporters (ko02010), and cutin, suberine, and wax biosynthesis (ko00073) (Figure S2A). This pattern demonstrates that the bacterial broth specifically activates the biosynthesis of antimicrobial secondary metabolites and components of physical barriers, alongside transmembrane transport processes. Conversely, the down-regulated DEGs were significantly enriched in distinct primary metabolic and cellular processing pathways, including protein processing in endoplasmic reticulum (ko04141), glutathione metabolism (ko00480), and starch and sucrose metabolism (ko00500) (Figure S2B). Collectively, these results indicate that the broth alone induces a transcriptional state of preparedness in wheat, characterized by the specific up-regulation of core defense and biosynthesis pathways coupled with a concurrent adjustment in primary metabolism and cellular homeostasis, aligning with the classic phenomenon of defense priming.

Transcriptional reshaping upon pathogen challenge of the primed host (Bv vs. BvFg). To define the response when a primed host encounters the pathogen, we analyzed the transition to combined stress (Bv vs. BvFg), which revealed 10,286 DEGs (Figure 4B; Table S9). Directional KEGG enrichment analysis (Figure S3, Table S9) revealed a focused and amplified defense response. The up-regulated DEGs were intensely enriched in a suite of defense-specific pathways, most notably the MAPK signaling pathway—plant (ko04016), phenylalanine metabolism (ko00360), plant–pathogen interaction (ko04626), and glutathione metabolism (ko00480) (Figure S3A). This demonstrates that the pre-primed state drives a potent up-regulation of defense signal transduction, amino acid metabolism linked to defense, pathogen recognition systems, and antioxidant activity. In contrast, the down-regulated DEGs were enriched in biosynthetic pathways, including cutin, suberine, and wax biosynthesis (ko00073) and fatty acid metabolism (ko01212) (Figure S3B). This result indicates that pathogen challenge in a primed host triggers a dual transcriptional strategy: a strong, specific amplification of induced defense and signaling pathways, coupled with a continued modulation of lipid and secondary cell wall-related metabolism. This coordinated shift underscores the efficacy of priming, enabling the host to rapidly mobilize specialized defense resources upon pathogen recognition.

Taken together, the transcriptomic analyses of the CK vs. Bv and Bv vs. BvFg comparisons delineate a coherent, two-phase mechanism by which *B. velezensis* 20507 fermentation broth primes and potentiates the wheat defense system. Initially, in the absence of the pathogen, the broth induces a transcriptional state of preparedness (defense priming). This state is characterized not by a broad up-regulation of all defense pathways, but by a targeted pre-activation of specific biosynthesis and transport functions (e.g., phenylpropanoid and cutin/suberine/wax biosynthesis, ABC transporters) alongside adjustments in primary metabolism and cellular homeostasis (Figure S2). Subsequently, when this primed plant encounters *F. graminearum*, the pre-configured transcriptional landscape enables a dramatically amplified and focused defense response. The transcriptional changes are intensively channeled into the potent up-regulation of a core suite of direct defense and signaling pathways, including MAPK signaling, plant–pathogen interaction, and associated metabolism (e.g., phenylalanine, glutathione) (Figure S3A), while continuing to modulate biosynthetic processes related to lipid and cell wall components (Figure S3B). This layered strategy—initial broad-spectrum preparedness followed by pathogen-triggered, targeted potentiation—explains how the bacterial broth transitions the host from a state of metabolic and defensive readiness to a state of effective, specialized resistance, providing a molecular blueprint for its biocontrol efficacy against FHB.

### 3.9. qRT-PCR Validation of RNA-Seq Data

**Figure 7:**
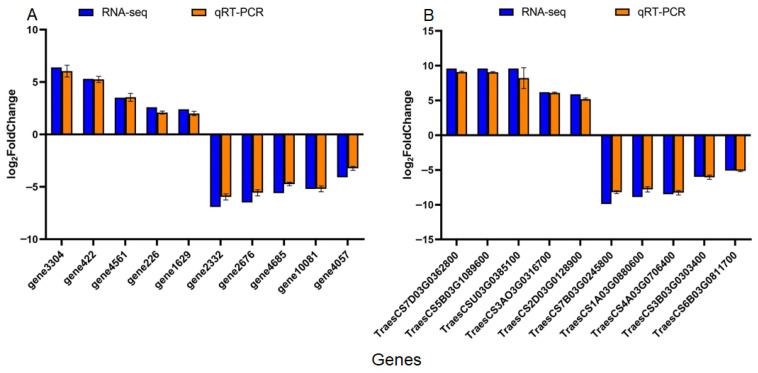
Validation of RNA-seq data by qRT-PCR analysis. (**A**) Relative expression levels of ten *Fusarium graminearum* differentially expressed genes (DEGs) from the CK vs. Fg comparison. (**B**) Relative expression levels of ten wheat (*Triticum aestivum*) DEGs from the Fg vs. BvFg comparison. Gene labels correspond to the standard *F. graminearum*PH-1 identifiers (FGSG numbers) and wheat genome identifiers (Traes IDs), respectively. Data are presented as mean ± SD (n = 3). The complete list of validated genes with their functional annotations is provided in Table 1.

To validate the reliability of the RNA-seq data, we performed quantitative real-time PCR (qRT-PCR) on 20 differentially expressed genes (DEGs)—10 from wheat (*T. aestivum*) and 10 from *F. graminearum*—all selected from the most significantly enriched pathways. The chosen wheat DEGs represent key processes identified in the transcriptome: one group comprised stress-response-related genes (e.g., genes involved in carbon fixation and the phosphoenolpyruvate carboxylase gene), which were markedly up-regulated after inoculation with *F. graminearum*; the other group consisted of genes associated with the suppression of basal metabolism (e.g., genes participating in cell-wall organization and pectin biosynthesis), which were significantly down-regulated upon *F. graminearum* infection. As shown, the relative expression trends of DEGs detected by qRT-PCR exhibited high consistency with the RNA-seq results in both the CK vs. Fg (Figure 7A) and Fg vs. BaFg (Figure 7B) comparisons. A significant positive correlation (R^2^ > 0.85, *p* < 0.001) was observed between the qRT-PCR and RNA-seq datasets. This strong correlation provides robust support for the accuracy and reliability of the transcriptomic data, particularly validating the expression patterns of genes related to defense priming (e.g., enhanced carbon fixation) and stress mitigation (e.g., reprogramming of basal metabolism) in wheat under *F. graminearum* challenge. Together, these results establish a solid data foundation for further in-depth exploration of the molecular mechanisms underlying wheat–*F. graminearum* interactions.

**Table 1:** Twenty selected genes validated by qRT-PCR and their functional annotation.

Gene	Annotation	Species
gene3304 (FGSG_03147)	Isocitrate lyase	*F. graminearum*
gene422 (FGSG_10694)	Hypothetical protein—Function unknown	*F. graminearum*
gene4561 (FGSG_04451)	Malate synthase	*F. graminearum*
gene226 (FGSG_00026)	Cystathionine beta-lyase	*F. graminearum*
gene1629 (FGSG_01373)	Hypothetical protein—Function unknown	*F. graminearum*
gene2332 (FPSE_05743)	Hypothetical protein—Function unknown	*F. graminearum*
gene2676 (FGSG_02597)	Copper amine oxidase 1	*F. graminearum*
gene4685 (FGSG_08596)	Hypothetical protein—Function unknown	*F. graminearum*
gene10081 (FGSG_13878)	Hypothetical protein—Function unknown	*F. graminearum*
gene4057 (FGSG_13783)	Hypothetical protein—Function unknown	*F. graminearum*
TraesCS7D03G0362800	Pathogenesis-related protein PRB1-3	*T. aestivum*
TraesCS5B03G1089600	Unnamed protein product—Function unknown	*T. aestivum*
TraesCSU03G0385100	Pathogenesis-related protein 1-8—A marker protein for plant defense responses	*T. aestivum*
TraesCS3A03G0316700	Phosphoenolpyruvate carboxylase 1-like	*T. aestivum*
TraesCS2D03G0128900	Ribulose bisphosphate carboxylase small chain	*T. aestivum*
TraesCS7B03G0245800	Unnamed protein product—Function unknown	*T. aestivum*
TraesCS1A03G0880600	Endoglucanase 3	*T. aestivum*
TraesCS4A03G0706400	Putative non-cyanogenic beta-glucosidase	*T. aestivum*
TraesCS3B03G0303400	Unnamed protein product—Function unknown	*T. aestivum*
TraesCS6B03G0811700	Beta-D-xylosidase 3-like	*T. aestivum*

### 3.10. Bioassay-Guided Identification of Key Antifungal Metabolites in the Fermentation Broth of B. velezensis 20507

**Figure 8:**
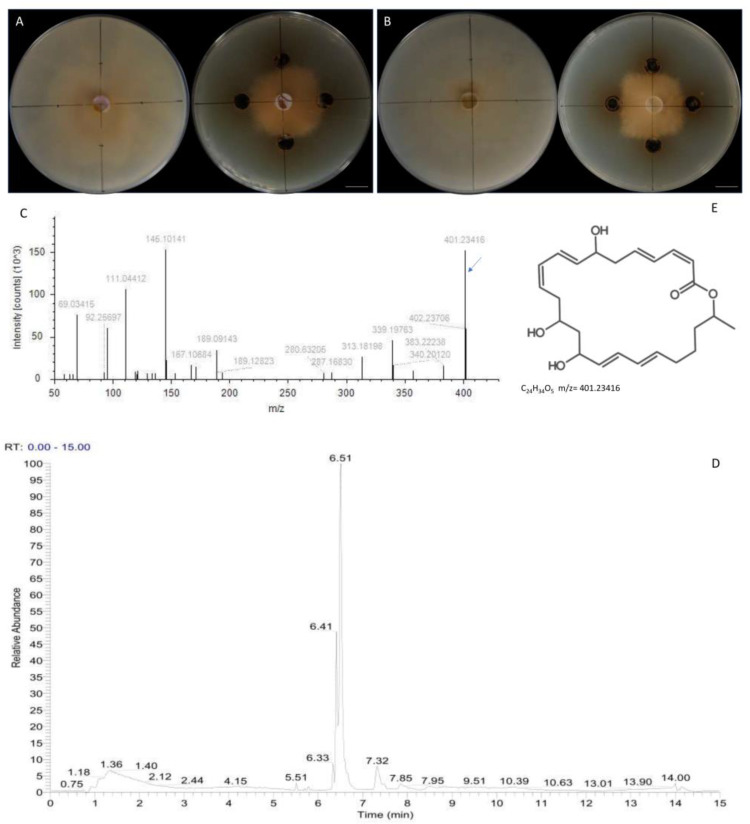
Identification and functional validation of key antifungal metabolites from *Bacillus velezensis* 20507 fermentation broth. (**A**) In vitro antagonistic assay of fraction Fr A (containing Macrolactin A as the major component) against *F. graminearum*. (**B**) In vitro antagonistic assay of fraction Fr B (lipopeptide-enriched) against *F. graminearum*. (**C**) High-resolution MS/MS fragmentation spectrum of the deprotonated molecule ([M–H]^−^) of Macrolactin A acquired in negative ion mode. The peak corresponding to Macrolactin A is indicated by an arrow. (**D**) Base peak chromatogram (BPC) of bioactive fraction Fr A in negative ion mode. (**E**) Molecular structure of Macrolactin A (C_24_H_34_O_5_). Scale bars: 1.0 cm (**A**,**B**).

To systematically identify the antifungal compounds responsible for the observed biocontrol effects, a bioassay-guided fractionation strategy was employed. The *Bacillus velezensis* 20507 fermentation broth was concentrated 10-fold and separated by preparative high-performance liquid chromatography (HPLC), yielding two bioactive fractions: Fr A (HPLC retention time 12–14 min) and Fr B (HPLC retention time 18–20 min). Both fractions consistently exhibited strong antifungal activity against *F. graminearum* in plate assays (Figure 8A,B).

The major constituent of Fr A was unambiguously identified as the macrolide Macrolactin A (C_24_H_34_O_5_, molecular mass ~402.24 Da) through untargeted LC-MS/MS analysis and database matching. Key evidence included: (i) an exact mass match for the deprotonated molecule ([M–H]^−^, observed *m*/*z* 401.2342, calculated: 401.2333, error < 3 ppm), (ii) a characteristic chromatographic retention time (Rt = 6.51 min), and (iii) a high-resolution MS/MS fragmentation spectrum whose key fragments matched the documented pattern of Macrolactin A (Figure 8C). Database searches (e.g., mzCloud, ChemSpider) confirmed Macrolactin A as the top match. The base peak chromatogram of Fr A showed a single dominant peak corresponding to this compound (Figure 8D), and its molecular structure is presented in Figure 8E.

In contrast, Fr B comprised a more complex mixture, as detailed in Table S2. Semi-quantitative analysis of the 47 detected compounds, which span different confidence levels of identification (Levels 2 and 3 per the Metabolomics Standards Initiative guidelines), indicated that lipopeptides and related variants were the most abundant constituents. Notably, key high-abundance compounds such as difficidin, surfactin C15, and bacillomycin D were identified with Level 2 confidence (putative annotation based on diagnostic MS/MS spectral match).

Antifungal bioassays confirmed that both purified fractions retained potent activity, with inhibition rates of approximately 62% (Fr A) and 60% (Fr B). Given that Macrolactin A is the principal component of Fr A and possesses well-documented antimicrobial properties, we propose it serves as a key direct antagonistic metabolite in the fermentation broth.

Collectively, these results demonstrate that the biocontrol efficacy of *B. velezensis* 20507 is mediated by a diverse suite of bioactive metabolites. Macrolactin A is identified as a principal antifungal agent, while the lipopeptide-enriched fraction Fr B also contributes substantially to pathogen suppression. This multi-component chemical arsenal underpins the complex direct antagonism employed by this strain.

## 4. Discussion

The development of effective and sustainable biocontrol strategies is crucial for managing Fusarium head blight (FHB), a disease that poses a persistent threat to global wheat production and food safety. In this study, we have provided integrated, multi-omics evidence that the fermentation broth of *Bacillus velezensis* 20507 functions as a potent biocontrol agent against *F. graminearum* through a coordinated dual mechanism of action, involving direct fungal antagonism and host defense potentiation. This comprehensive perspective validates the dual-pronged mechanistic hypothesis proposed at the outset of our investigation. Our findings bridge the specific chemical arsenal of a biocontrol bacterium with its functional consequences for both the pathogen and the host plant, offering a mechanistic framework that underscores the strain’s potential for sustainable FHB management.

### 4.1. Direct Antagonism: Disrupting Fungal Core Metabolism

The first pillar of the biocontrol mechanism employed by *B. velezensis* 20507 fermentation broth is its direct and potent antagonistic activity against *F. graminearum*, a hallmark of many effective biocontrol agents. Our in vitro assays robustly confirmed this activity, demonstrating that the broth forms distinct inhibition zones and suppresses mycelial radial growth by 59.8% (Figure 1A,B). This aligns with the well-established role of *Bacillus* spp. as prolific producers of diverse antimicrobial compounds, which form the chemical basis of their antagonism [36,37,38,39].

Our integrated multi-omics approach provides a molecular blueprint linking this chemical arsenal to its functional consequences. Metabolomic profiling identified a suite of bioactive compounds, including lipopeptides (e.g., surfactins, bacillomycin D) and the polyketide macrolide Macrolactin A (Figure 8, Table S2). Crucially, dual RNA-seq analysis of the *in planta* interaction revealed the profound impact of these metabolites on the pathogen. A drastic, ~68-fold reduction in reads uniquely mapping to the *F. graminearum* genome in co-inoculated (BvFg) versus pathogen-only (Fg) samples provided direct, sequencing-level evidence for a severe, global suppression of fungal transcriptional activity (Table S4). Subsequent differential expression analysis pinpointed the specific metabolic targets of this suppression: 1510 genes were differentially expressed, with a strong bias towards down-regulation. Functional enrichment revealed that genes involved in core carbohydrate metabolism (e.g., carbon metabolism, starch and sucrose metabolism) and cell wall-related processes (e.g., polysaccharide catabolic process) were significantly down-regulated (Figure 3B–D).

This coordinated disruption of central metabolic and cellular maintenance pathways provides direct experimental validation for the first component of our hypothesis. The down-regulation of carbohydrate metabolism genes likely cripples the pathogen’s energy (ATP) production and carbon skeleton supply, fundamentals for growth, toxin biosynthesis, and virulence [2]. Simultaneously, the suppression of cell wall-related processes may reflect a compromised ability to maintain cellular integrity, potentially synergizing with the action of membrane-targeting lipopeptides identified in the broth [38,39]. While our data did not indicate significant up-regulation of *B. velezensis*-encoded extracellular hydrolytic enzymes—a mechanism employed by some biocontrol agents [37,40]—the observed fungal transcriptional profile is indicative of a profound metabolic paralysis.

This multi-target mode of action, simultaneously impairing several essential metabolic hubs and cellular structures, represents a significant advantage over single-site chemical fungicides. By deploying a cocktail of bioactive metabolites that collectively disrupt fundamental processes, *B. velezensis* 20507 exerts a strong selective pressure that is less likely to be overcome by a single resistance mutation in the pathogen. This strategy aligns with the coordinated mechanisms that underpin the robustness of biological control systems [36], forming a solid foundation for the direct antagonism pillar of its biocontrol efficacy.

### 4.2. Host Modulation: From Priming to Amplified Defense

The second, equally crucial pillar of the biocontrol mechanism is the active modulation of the wheat host’s physiology. Our transcriptomic data delineate a sophisticated, two-phase interaction, showing that *B. velezensis* 20507 broth not only directly antagonizes the pathogen but also orchestrates a state of enhanced host readiness, confirming the second arm of our hypothesis regarding defense priming and metabolic reprogramming.

In the absence of the pathogen, the broth alone initiated a transcriptional state of defense priming. Directional KEGG analysis (CK vs. Bv) showed this state was characterized by the up-regulation of key defense and biosynthesis pathways, most notably phenylpropanoid biosynthesis, ABC transporters, and cutin, suberine, and wax biosynthesis, alongside the down-regulation of pathways related to primary metabolism and cellular processing, such as protein processing in the endoplasmic reticulum, glutathione metabolism, and starch and sucrose metabolism (Figure S2). This reprogramming aligns with the classic phenomenon of microbially induced resistance [41,42].

This primed state was not static. Upon pathogen challenge, it enabled a dramatically amplified and targeted defense response (Bv vs. BvFg). The pre-primed host intensely up-regulated a core suite of defense and signaling pathways, including MAPK signaling, phenylalanine metabolism, plant–pathogen interaction, and glutathione metabolism, to levels suggesting an amplified response (Figure S3A). This indicates efficient channeling of resources into a potent defense output, likely amplifying hormone-mediated signaling [42,43], supported by the continued modulation of biosynthetic pathways such as cutin, suberine, and wax biosynthesis and fatty acid metabolism (Figure S3B)**.**

While we acknowledge that the overall reduction in host transcript abundance in pathogen-only (Fg) samples could be influenced by wound-associated tissue damage, multiple lines of evidence support that our data predominantly reflect specific, biologically meaningful reprogramming. Firstly, high RNA integrity (RIN ≥ 7.0) across samples argues against massive, non-specific degradation. Secondly, and more critically, the directional KEGG enrichment analyses revealed coherent and contrasting transcriptional signatures. Pathogen infection alone (CK vs. Fg) instigated a dichotomous response: it activated canonical defense pathways such as the MAPK signaling pathway and phenylpropanoid biosynthesis (Figure 6A), while concurrently suppressing a wide array of fundamental biosynthetic and energy metabolism pathways, including amino acid, carbon, and glycan metabolism (Figure 6B). This pattern is indicative of a pathogenic strategy that disrupts host metabolic homeostasis. The application of *B. velezensis* 20507 fermentation broth during co-inoculation (BvFg vs. Fg) effectively counteracted and reshaped this pathogenic reprogramming. It not only maintained or potentiated the up-regulation of key defense-related pathways (Figure 5A) but also orchestrated a distinct modulation of host primary metabolism. Specifically, the broth induced the down-regulation of pathways such as glutathione metabolism, amino acid biosynthesis, and carbon metabolism (Figure 5B), which differs from the broader metabolic suppression pattern caused by the pathogen alone. This coordinated transcriptional shift suggests that the biocontrol agent facilitates a strategic reallocation of host resources, diverting them from growth-oriented biosynthetic processes to sustain and enhance the activated defense program, thereby establishing a more effective resistance state against *F. graminearum*.

The observed host modulation exemplifies induced resistance, a core biocontrol mechanism [36,41]. It operates in concert with direct antagonism. Identified metabolites like lipopeptides and Macrolactin A likely serve a dual role: directly inhibiting the pathogen while acting as elicitors for induced systemic resistance [38,44]. This coordinated dual action—direct inhibition coupled with host defense potentiation—creates a robust, multi-layered defense barrier that underpins the strain’s efficacy against FHB. Our finding that the host’s response involves both broad-spectrum priming and a pathogen-triggered, amplified defense wave is consistent with the genotype-specific yet converging defense strategies reported in diverse wheat-FHB pathosystems [45].

### 4.3. Chemical Basis of Antagonism and Integration of Mechanisms

Our metabolomic analysis successfully bridges the observed biocontrol phenotypes with their underlying chemical drivers. We identified a diverse suite of antimicrobials, with the macrolide Macrolactin A as the principal component of the bioactive fraction Fr A, and a mixture of lipopeptides (e.g., difficidin, surfactins) enriching fraction Fr B (Figure 8, Table S2). This chemical repertoire provides the direct agents likely responsible for both the severe transcriptional and metabolic disruption observed in the pathogen (*in planta*; Figure 3) and for triggering the induced systemic resistance (ISR)-like priming response in the host [41,46].

Macrolactin A is a 24-membered macrolide with broad-spectrum antimicrobial activity. While its precise mode of action against *F. graminearum* warrants further study, the literature suggests mechanisms—including membrane disruption and interference with macromolecular synthesis—that provide a plausible basis for the widespread transcriptional down-regulation and metabolic paralysis we documented [47,48,49]. The co-produced lipopeptides are well-known to target fungal membranes and cellular integrity, likely acting in concert with Macrolactin A to exert the potent, direct antagonism [38,39].

This integrated multi-omics perspective coherently resolves the proposed hypothesis. The identified metabolites directly debilitate the fungus, explaining the collapse of its core metabolism (Figure 3). Concurrently, these or other broth components serve as elicitors, reprogramming the host: they prime defense pathways in the absence of the pathogen (Figure S2) and enable an amplified, targeted defense response upon challenge (Figure S3), thereby countering the pathogen’s own disruptive reprogramming (Figure 5 and Figure 6). The high correlation between RNA-seq and qRT-PCR data (Figure 7) solidifies the reliability of these transcriptomic insights. Collectively, our findings demonstrate that the biocontrol efficacy of *B. velezensis* 20507 stems from a coordinated dual mechanism: a direct, multi-component chemical attack on the pathogen, coupled with the sophisticated pre-activation and potentiation of the host’s innate defense system, a strategy critical for effective biological control [36].

## 5. Conclusions

**Figure 9:**
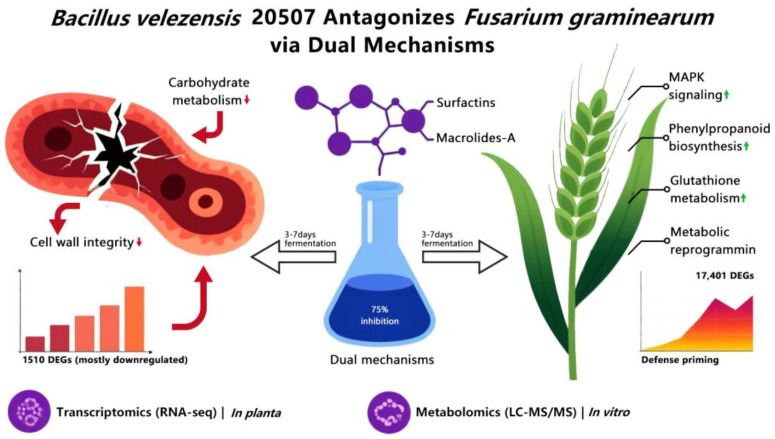
Dual antagonistic mechanism of *Bacillus velezensis* 20507 fermentation broth against Fusarium head blight. Downward and upward arrows indicate downregulated and upregulated gene expression, respectively.

Integrated multi-omics analyses in this study revealed that *B*. *velezensis* 20507 combats Fusarium head blight (FHB) through a synergistic dual mechanism, as summarized in the proposed model (Figure 9). This mechanism comprises: (1) direct antifungal action, where metabolites such as lipopeptides and the macrolide Macrolactin A disrupt the core metabolism of *F*. *graminearum*, thereby inhibiting its growth, and (2) host transcriptional reprogramming, whereby the bacterial fermentation broth actively reshapes the wheat defense response. Transcriptomic analyses indicate that the biocontrol agent does not simply reverse the pathogen-induced dysregulation but initiates a unique and optimized reprogramming. During co-inoculation, it specifically up-regulates pathways associated with energy reallocation, phytohormone signaling, and cellular maintenance, while concurrently down-regulating a distinct set of primary metabolic pathways. This coordinated shift reprograms the host from a state of pathogenic stress and metabolic imbalance into an optimized state characterized by reconfigured resource allocation and enhanced defense. Consequently, the biocontrol efficacy stems from the synergistic dual action of direct pathogen suppression and host defense reprogramming. This work deciphers the molecular and chemical blueprint underlying the biocontrol activity of *B. velezensis* 20507, providing a scientific foundation for its sustainable application against FHB. It should be noted that the seedling stem-wounding inoculation method used here, while controllable, represents an artificial infection route. Future validation under more field-relevant conditions (e.g., spray inoculation on spikes) is warranted to assess the translational potential of these findings.

## Data Availability

The raw transcriptome sequencing data of soybean presented in this study are openly available in the national center for biotechnology information (NCBI) database, reference number PRJNA1152285. The untargeted metabolomics mass spectrometry raw data have been deposited in Zenodo at https://doi.org/10.5281/zenodo.19279834.

## Supplementary Materials

The following supporting information can be downloaded at: https://www.mdpi.com/article/10.3390/microorganisms14051039/s1, Figure S1: Time-course assessment of antifungal metabolite production; Figure S2: Kyoto Encyclopedia of Genes and Genomes (KEGG) pathway enrichment analysis of the wheat transcriptome modulated by *Bacillus velezensis* 20507 fermentation broth; Figure S3: Kyoto Encyclopedia of Genes and Genomes (KEGG) pathway enrichment analysis of the wheat transcriptome modulated by pathogen challenge under biocontrol priming; Table S1: Primer sequences employed in qRT-PCR; Table S2: Chemical Constituents of *B. velezensis* 20507 Fermentation Broth (LC-MS); Table S3: Sequencing data statistics; Table S4: Read mapping efficiency to the *Fusarium graminearum* genome; Table S5: Read mapping efficiency to the *Triticum aestivum* genome; Table S6: Differentially expressed genes associated with *F*. *graminearum* in the Fg vs. BvFg comparison; Table S7: Differentially expressed genes associated with *Triticum aestivum* in the CK vs. Fg comparison; Table S8: Differentially expressed genes associated with *Triticum aestivum* in the CK vs. Bv comparison; Table S9: Differentially expressed genes associated with *Triticum aestivum* in the Bv vs. BvFg comparison.

## References

[1] Moonjely S., Ebert M., Paton-Glassbrook D., Noel Z.A., Roze L., Shay R., Watkins T., Trail F. (2023). Update on the state of research to manage *Fusarium* head blight. Fungal Genet. Biol..

[2] Khan M.K., Pandey A., Athar T., Choudhary S., Deval R., Gezgin S., Hamurcu M., Topal A., Atmaca E., Santos P.A. (2020). *Fusarium* head blight in wheat: Contemporary status and molecular approaches. 3 Biotech.

[3] Merhej J., Richard-Forget F., Barreau C. (2011). Regulation of trichothecene biosynthesis in *Fusarium*: Recent advances and new insights. Appl. Microbiol. Biotechnol..

[4] Khan R., Anwar F., Ghazali F.M. (2024). A comprehensive review of mycotoxins: Toxicology, detection, and effective mitigation approaches. Heliyon.

[5] Johns L.E., Bebber D.P., Gurr S.J., Brown N.A. (2022). Emerging health threat and cost of *Fusarium* mycotoxins in European wheat. Nat. Food.

[6] Zhang L., Jia X., Chen C., Zhou M. (2013). Characterization of carbendazim sensitivity and trichothecene chemotypes of *Fusarium graminearum* in Jiangsu Province of China. Physiol. Mol. Plant Pathol..

[7] Tang G., Chen Y., Xu J.R., Kistler H.C., Ma Z. (2018). The fungal myosin I is essential for *Fusarium* toxisome formation. PLoS Pathog..

[8] Audenaert K., Callewaert E., Höfte M., De Saeger S., Haesaert G. (2010). Hydrogen peroxide induced by the fungicide prothioconazole triggers deoxynivalenol (DON) production by *Fusarium graminearum*. BMC Microbiol..

[9] Marques L.N., Pizzutti I.R., Balardin R.S., Dos Santos I.D., Dias J.V., Stefanello M.T., Serafini P.T. (2017). Occurrence of mycotoxins in wheat grains exposed to fungicides on *Fusarium* head blight control in southern Brazil. J. Environ. Sci. Health Part B.

[10] Figueroa M., Hammond-Kosack K.E., Solomon P.S. (2018). A review of wheat diseases-a field perspective. Mol. Plant Pathol..

[11] Legrand F., Picot A., Cobo-Díaz J.F., Chen W., Le Floch G. (2017). Challenges facing the biological control strategies for the management of *Fusarium* Head Blight of cereals caused by *F. graminearum*. Biol. Control.

[12] Chen Y., Kistler H.C., Ma Z. (2019). *Fusarium graminearum* Trichothecene Mycotoxins: Biosynthesis, Regulation, and Management. Annu. Rev. Phytopathol..

[13] Bencheikh A., Belabed I., Rouag N. (2024). *Fusarium* head blight of wheat: Current knowledge on associated species and their mycotoxins, pathogenicity diversity, and management strategies. Australas. Plant Pathol..

[14] Wegulo S.N., Baenziger P.S., Hernandez Nopsa J., Bockus W.W., Hallen-Adams H. (2015). Management of *Fusarium* head blight of wheat and barley. Crop Prot..

[15] Mutlu A., Kaspar C., Becker N., Bischofs I.B. (2020). A spore quality-quantity trade off favors diverse sporulation strategies in *Bacillus subtilis*. ISME J..

[16] Piggot P.J., Hilbert D.W. (2004). Sporulation of *Bacillus subtilis*. Curr. Opin. Microbiol..

[17] Ntushelo K., Ledwaba L.K., Rauwane M.E., Adebo O.A., Njobeh P.B. (2019). The mode of action of *Bacillus* species against *Fusarium graminearum*, tools for investigation, and future prospects. Toxins.

[18] Yi Y., Luan P., Fan M., Wu X., Sun Z., Shang Z., Yang Y., Li C. (2024). Antifungal efficacy of *Bacillus amyloliquefaciens*ZK-9 against *Fusarium graminearum* and analysis of the potential mechanism of its lipopeptides. Int. J. Food Microbiol..

[19] Yu C., Liu X., Zhang X., Zhang M., Gu Y., Ali Q., Mohamed M.S.R., Xu J., Shi J., Gao X. (2021). Mycosubtilin produced by *Bacillus subtilis* ATCC6633 inhibits growth and mycotoxin biosynthesis of *Fusarium graminearum* and *Fusarium verticillioides*. Toxins.

[20] Zhao Y., Selvaraj J.N., Xing F., Zhou L., Wang Y., Song H., Tan X., Sun L., Sangare L., Folly Y.M. (2014). Antagonistic action of *Bacillus subtilis* strain SG6 on *Fusarium graminearum*. PLoS ONE.

[21] Kim K., Lee Y., Ha A., Kim J.I., Park A.R., Yu N.H., Son H., Choi G.J., Park H.W., Lee C.W. (2017). Chemosensitization of *Fusarium graminearum* to chemical fungicides using cyclic lipopeptides produced by *Bacillus amyloliquefaciens* strain JCK-12. Front. Plant Sci..

[22] Palazzini J., Reynoso A., Yerkovich N., Zachetti V., Ramírez M., Chulze S. (2022). Combination of *Bacillus velezensis* RC218 and chitosan to control *Fusarium* head blight on bread and durum wheat under greenhouse and field conditions. Toxins.

[23] Cheng Y., Lou H., He H., He X., Wang Z., Gao X., Liu J. (2024). Genomic and biological control of *Sclerotinia sclerotiorum* using an extracellular extract from *Bacillus velezensis* 20507. Front. Microbiol..

[24] Chen S., Zhou Y., Chen Y., Gu J. (2018). fastp: An ultra-fast all-in-one FASTQ preprocessor. Bioinformatics.

[25] Kan L., Liao Q., Chen Z., Wang S., Ma Y., Su Z., Zhang L. (2021). Dynamic transcriptomic and metabolomic analyses of *Madhuca pasquieri* (Dubard) H. J. Lam during the post-germination stages. Front. Plant Sci..

[26] Zhang Y., Song L., Hou L., Cao Z., Vongsangnak W., Zhu G., Xu Q., Chen G. (2021). Dual transcriptomic analyses unveil host-pathogen interactions between *Salmonella enterica* serovar Enteritidis and laying ducks (*Anas platyrhynchos*). Front. Microbiol..

[27] Martínez-Padrón H.Y., Herrera-Mayorga V., Paredes-Sánchez F.A., Lara-Ramírez E.E., Torres-Castillo J.A., Rodríguez-Herrera R., López-Santillán J.A., Osorio-Hernández E. (2023). In vitro evaluation of the antagonistic activity of native strains of *Trichoderma* spp. against *Fusarium* spp. J. Environ. Sci. Health Part B.

[28] Yassin M.T., Mostafa A.A.F., Al-Askar A.A. (2021). In vitro antagonistic activity of *Trichoderma harzianum* and *T. viride* strains compared to carbendazim fungicide against the fungal phytopathogens of *Sorghum bicolor*(L.) Moench. Egypt. J. Biol. Pest. Control.

[29] Kim D., Langmead B., Salzberg S.L. (2015). HISAT: A fast spliced aligner with low memory requirements. Nat. Methods.

[30] Pertea M., Pertea G.M., Antonescu C.M., Chang T.C., Mendell J.T., Salzberg S.L. (2015). StringTie enables improved reconstruction of a transcriptome from RNA-seq reads. Nat. Biotechnol..

[31] Shumate A., Wong B., Pertea G., Pertea M. (2022). Improved transcriptome assembly using a hybrid of long and short reads with StringTie. PLoS Comput. Biol..

[32] Liu S., Wang Z., Zhu R., Wang F., Cheng Y., Liu Y. (2021). Three differential expression analysis methods for RNA sequencing: Limma, EdgeR, DESeq2. J. Vis. Exp..

[33] Xu S., Hu E., Cai Y., Xie Z., Luo X., Zhan L., Tang W., Wang Q., Liu B., Wang R. (2024). Using cluster Profiler to characterize multiomics data. Nat. Protoc..

[34] Yu G. (2024). Thirteen years of cluster Profiler. Innovation.

[35] Schmittgen T.D., Livak K.J. (2008). Analyzing real-time PCR data by the comparative C(T) method. Nat. Protoc..

[36] Köhl J., Kolnaar R., Ravensberg W.J. (2019). Mode of action of microbial biological control agents against plant diseases: Relevance beyond efficacy. Front. Plant Sci..

[37] Matarese F., Sarrocco S., Gruber S., Seidl-Seiboth V., Vannacci G. (2012). Biocontrol of *Fusarium* head blight: Interactions between *Trichoderma* and mycotoxigenic *Fusarium*. Microbiology.

[38] Huang Y., Zhang X., Xu H., Zhang F., Zhang X., Yan Y., He L., Liu J. (2022). Isolation of lipopeptide antibiotics from *Bacillus siamensis*: A potential biocontrol agent for *Fusarium graminearum*. Can. J. Microbiol..

[39] Dunlap C.A., Schisler D.A., Price N.P., Vaughn S.F. (2011). Cyclic lipopeptide profile of three *Bacillus subtilis* strains; antagonists of *Fusarium* head blight. J. Microbiol..

[40] Yeo Y.J., Park A.R., Vuong B.S., Kim J.C. (2024). Biocontrol of *Fusarium* head blight in rice using *Bacillus velezensis* JCK-7158. Front. Microbiol..

[41] Yu Y., Gui Y., Li Z., Jiang C., Guo J., Niu D. (2022). Induced systemic resistance for improving plant immunity by beneficial microbes. Plants.

[42] Conrath U., Beckers G.J., Langenbach C.J., Jaskiewicz M.R. (2015). Priming for enhanced defense. Annu. Rev. Phytopathol..

[43] Meziane H., van der Sluis I., van Loon L.C., Höfte M., Bakker P.A. (2005). Determinants of *Pseudomonas putida* WCS358 involved in inducing systemic resistance in plants. Mol. Plant Pathol..

[44] Zhang N., Wang Z., Shao J., Xu Z., Liu Y., Xun W., Miao Y., Shen Q., Zhang R. (2023). Biocontrol mechanisms of *Bacillus*: Improving the efficiency of green agriculture. Microb. Biotechnol..

[45] Pan Y., Liu Z., Rocheleau H., Fauteux F., Wang Y., McCartney C., Ouellet T. (2018). Transcriptome dynamics associated with resistance and susceptibility against *Fusarium* head blight in four wheat genotypes. BMC Genom..

[46] Schisler D.A., Core A.B., Boehm M.J., Horst L., Krause C., Dunlap C.A., Rooney A.P. (2014). Population dynamics of the *Fusarium* head blight biocontrol agent *Cryptococcus flavescens* OH 182.9 on wheat anthers and heads. Biol. Control.

[47] Khondker A., Bider R.-C., Passos-Gastaldo I., Wright G.D., Rheinstädter M.C. (2021). Membrane interactions of non-membrane targeting antibiotics: The case of aminoglycosides, macrolides, and fluoroquinolones. Biochim. Biophys. Acta (BBA)—Biomembr..

[48] Krawczyk S.J., Leśniczak-Staszak M., Gowin E., Szaflarski W. (2024). Mechanistic Insights into Clinically Relevant Ribo-some-Targeting Antibiotics. Biomolecules.

[49] Shen J. (2025). The future of macrolide antibiotics: Modification and new discoveries. Theor. Nat. Sci..

