# Supplementary material for: Transcriptomics and Metabolomics Reveal the Antagonistic Mechanism of Bacillus velezensis 20507 Fermentation Broth Against Fusarium Head Blight Pathogen
Source: Microorganisms. 2026 May 3;14(5):1039. doi: 10.3390/microorganisms14051039 (PMC13209314; doi:10.3390/microorganisms14051039)
Supplement: Supplementary file 1 [file microorganisms-14-01039-s001.zip › Figure S1.pdf]

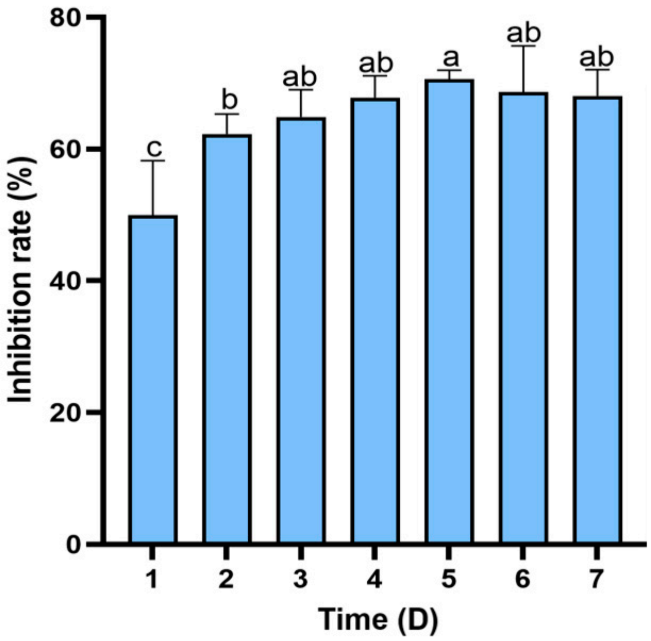

Figure S1. Time-Course Assessment of Antifungal Metabolite Production

Note: Different lowercase letters above the bars indicate statistically significant differences among the treatments as determined by one-way ANOVA followed by Duncan's multiple range test ($P < 0.05$). Data are presented as mean ± SD ($n = 3$).

The inhibition rate of the fermentation broth against *Fusarium graminearum* increased over time and reached a plateau at approximately 75% on day 5, with no further enhancement observed on days 6 and 7.
