# Supplementary material for: Transcriptomics and Metabolomics Reveal the Antagonistic Mechanism of Bacillus velezensis 20507 Fermentation Broth Against Fusarium Head Blight Pathogen
Source: Microorganisms. 2026 May 3;14(5):1039. doi: 10.3390/microorganisms14051039 (PMC13209314; doi:10.3390/microorganisms14051039)
Supplement: Supplementary file 1 [file microorganisms-14-01039-s001.zip › Figure S2.pdf]

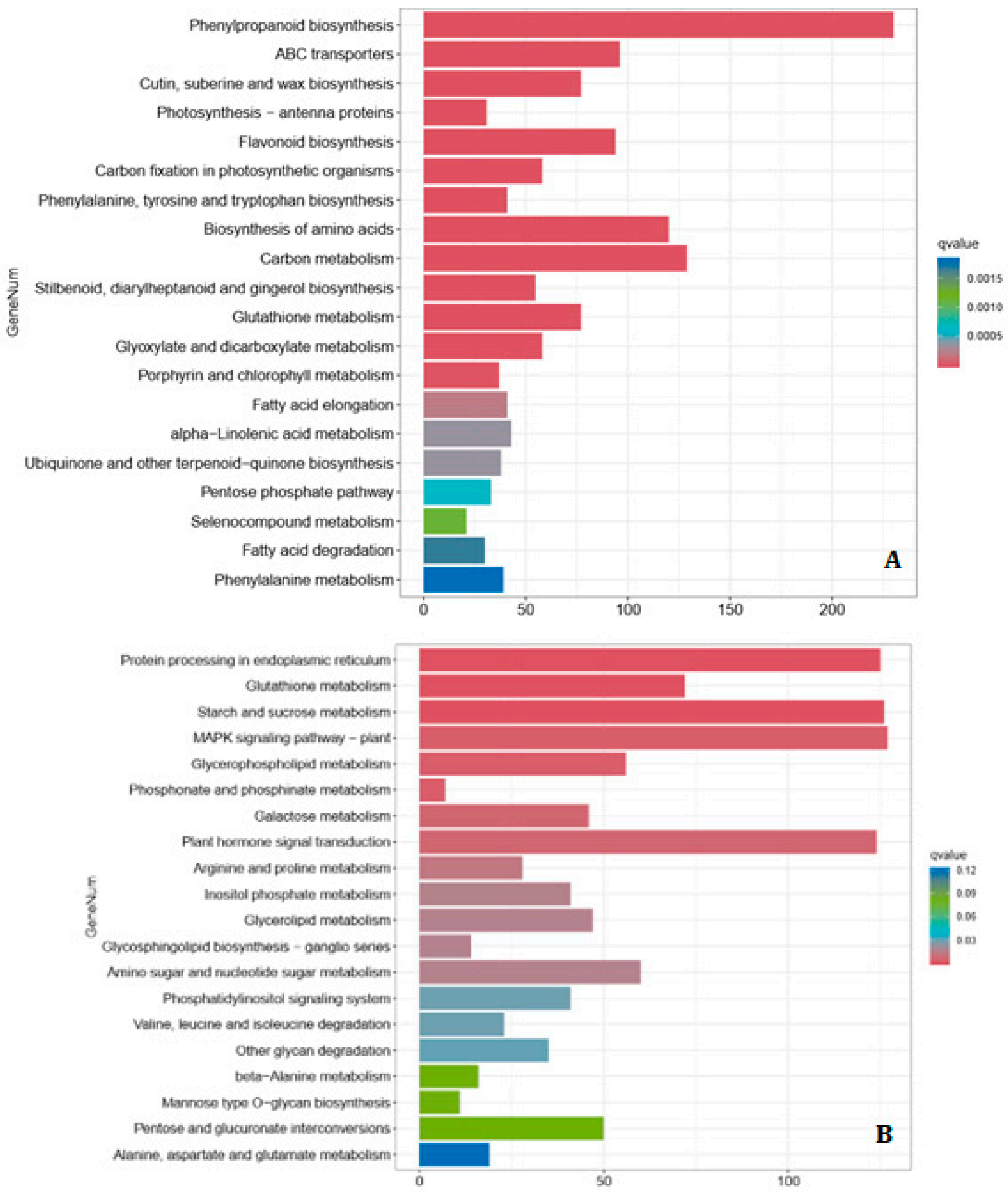

Figure S2. Kyoto Encyclopedia of Genes and Genomes (KEGG) pathway enrichment analysis of the wheat transcriptome modulated by *Bacillus velezensis*20507 fermentation broth. (A) Top enriched KEGG pathways for up-regulated differentially expressed genes (DEGs) in the CK vs. Bv comparison. (B) Top enriched KEGG pathways for down-regulated DEGs in the CK vs. Bv comparison. Treatments: CK, mock-treated control; Bv, treated with *B. velezensis*20507 fermentation broth. All RNA-seq reads from wheat samples were uniquely mapped to the *Triticum aestivum* reference genome.
