# Supplementary material for: Transcriptomics and Metabolomics Reveal the Antagonistic Mechanism of Bacillus velezensis 20507 Fermentation Broth Against Fusarium Head Blight Pathogen
Source: Microorganisms. 2026 May 3;14(5):1039. doi: 10.3390/microorganisms14051039 (PMC13209314; doi:10.3390/microorganisms14051039)
Supplement: Supplementary file 1 [file microorganisms-14-01039-s001.zip › Figure S3.pdf]

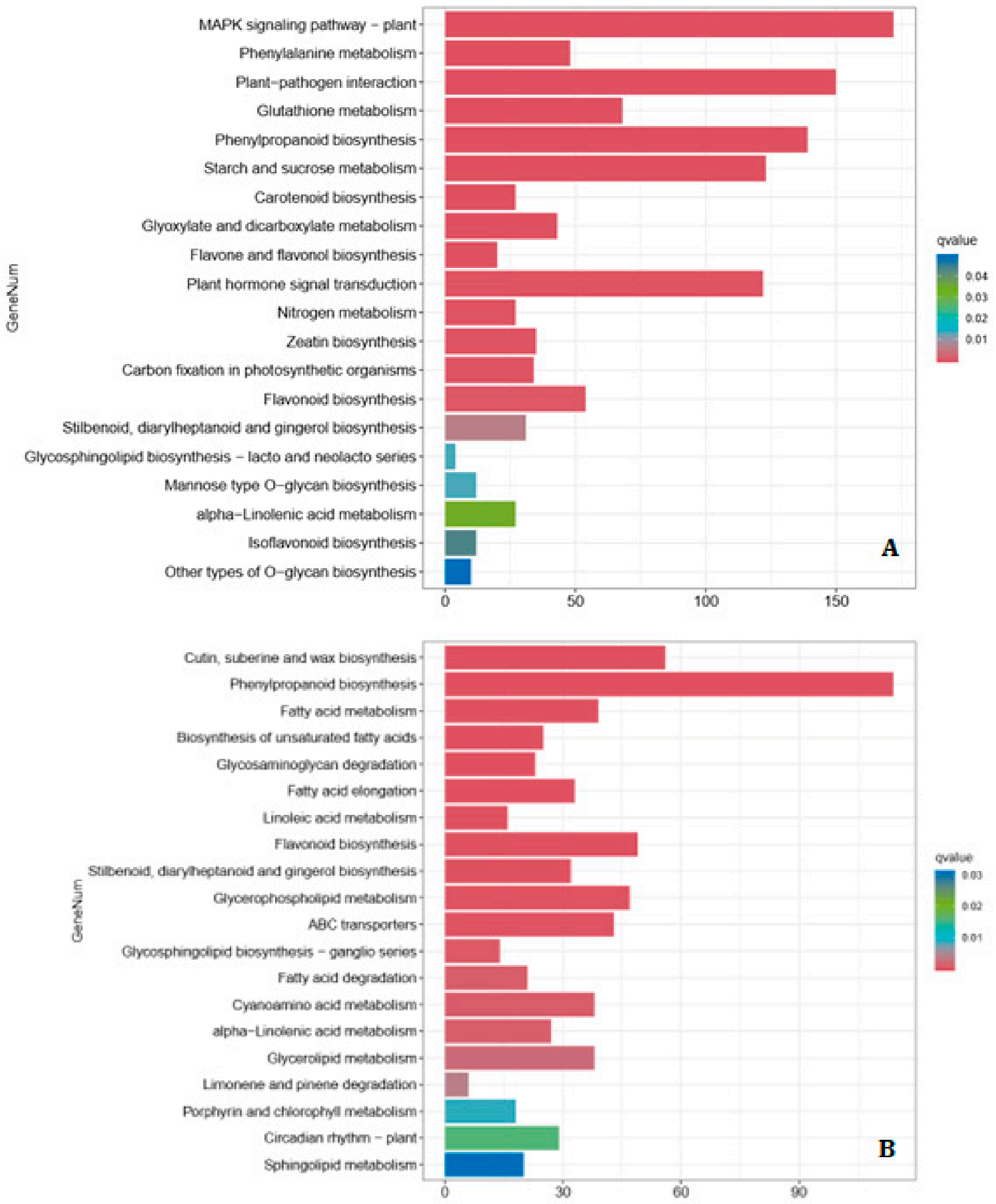

Figure S3. Kyoto Encyclopedia of Genes and Genomes (KEGG) pathway enrichment analysis of the wheat transcriptome modulated by pathogen challenge under biocontrol priming. (A) Top enriched KEGG pathways for up-regulated differentially expressed genes (DEGs) in wheat pre-treated with *B. velezensis*20507 fermentation broth and subsequently challenged with *F. graminearum*(BvFg) compared to the broth treatment alone (Bv). (B) Top enriched KEGG pathways for down-regulated DEGs in the BvFg vs. Bv comparison. This comparison reveals the specific transcriptional reprogramming in wheat induced by the pathogen in a host that was pre-primed by the biocontrol agent. All RNA-seq reads from wheat samples were uniquely mapped to the *Triticum aestivum* reference genome.
