# Supplementary material for: Transcriptomics and Metabolomics Reveal the Antagonistic Mechanism of Bacillus velezensis 20507 Fermentation Broth Against Fusarium Head Blight Pathogen
Source: Microorganisms. 2026 May 3;14(5):1039. doi: 10.3390/microorganisms14051039 (PMC13209314; doi:10.3390/microorganisms14051039)
Supplement: Supplementary file 1 [file microorganisms-14-01039-s001.zip › Table S1.pdf]

Table S1 Primer sequences employed in qRT-PCR

gene3304	XM_011324138.1.F：ATCGGTAACCCGCTCCAC
	XM_011324138.1.R：GCTGAAATTCCGCTCACG
gene422	XM_011317786.1.F：CTCGTCGTTGAAGGCACA
	XM_011317786.1.R：CGAGATCGTCGGAGTGAAG
gene4561	XM_011322723.1.F：CGTCCTCAGTTCTACCTCT
	XM_011322723.1.R：TGAAAGTAAATCTGTCCCTA
gene226	XM_011317549.1 .F：TCCTCTTGTCTTTCGCACCA
	XM_011317549.1 .R：CCTTCTCCCGCTTCTCCA
gene1629	XM_011319151.1.F：CACTGTACGACGATGGGTT
	XM_011319151.1.R：GTAGCGGTCAGCAGAGGT
gene2332	XM_011319939.1.F：CAAGCAACTCGGATAACTC
	XM_011319939.1.R：TCATCTCATCAACACCACC
gene2676	XM_0113203251.F：ACCCGACAGCGAAACTAT
	XM_011320325.1.R：GTATATGAATGTTGATTCAT
gene4685	XM_011322579.1.F：ACCCGACAGCGAAACTAT
	XM_011322579.1.R：CCGTAGCCTAAGCACTCA
gene10081	XM_011320711.1.F：TTCACCGCAGGGTTTATC
	XM_011320711.1.R：AACTTGACCGTGGGAATG
gene4057	XM_011323292.1.F：GCATTGCTCGCCATTCTC
	XM_011323292.1.R：TGAACTGACGGCCTGGAA
TraesCS7D03G0362800	XM_044587903.1.F：GACTACGACTACGGCTCCAAC
	XM_044587903.1.R：GAAGACGCCGAGGTTATTGT
TraesCS5B03G1089600	XM_044538026.1.F：TTCGTGGGTGTCGGAGAAG
	XM_044538026.1.R：ACCACCTGCGTGTAGTGCC
TraesCSU03G0385100	XM_044591036.1.F：TACGGGGAGAACCTCTACGG
	XM_044591036.1.R：TCTCCGACACCCACGAATT
TraesCS3A03G0316700	XM_044483418.1.F：TCAGAGCCAACTACGCCGAGAC
	XM_044483418.1.R：CGCCGTGATGTACGAGTCCC
TraesCS2D03G0128900	XM_044474098.1.F：CAGCAATGGCGGAAGGAT
	XM_044474098.1.R：GGGTGGCAAGTAAGACAAGG
TraesCS7B03G0245800	XM_044576429.1.F：CCTGTGCGTGGGAGTATGG

	XM_044576429.1.R：CCTGCTGGTAGAACAGATGTCG
TraesCS1A03G0880600	XM_044488437.1.F：GGCGTTCAGCTCGACGATG
	XM_044488437.1.R：CGGTGGCCTTGAGGAGGTAG
TraesCS4A03G0706400	XM_044509067.1.F：ATCAGGCAGCTCAGAAGG
	XM_044509067.1.R：ACCAGCCAACATGGAAGT
TraesCS3B03G0303400	XM_044491614.1.F：TGTTCTATGGACTGGACCTA
	XM_044491614.1.R：GGACTCGCAGCTAATGAC
TraesCS6B03G0811700	XM_044557577.1.F：CAAGGTCCATTCGTTGTCACTG
	XM_044557577.1.R：TGCATGGTTTGCCTCTGTAGTC
β-TUB	F：ACTCCAAGAACATGATGTG
	R：GATCCACTCGACGAAGTA
FgEF1A	F：GGCTTTCACCGACTACCCTCCTCT
	R：ACTTCTCGACGGCCTTGATGACAC
